# Leveraging Deep Learning, Grid Search, and Bayesian Networks to Predict Distant Recurrence of Breast Cancer

**DOI:** 10.3390/cancers17152515

**Published:** 2025-07-30

**Authors:** Xia Jiang, Yijun Zhou, Alan Wells, Adam Brufsky

**Affiliations:** 1 Department of Biomedical Informatics, University of Pittsburgh, Pittsburgh, PA 15206, USA; 2 Department of Pathology, University of Pittsburgh and Pittsburgh VA Health System, Pittsburgh, PA 15261, USA; 3 UPMC Hillman Cancer Center, Pittsburgh, PA 15232, USA; 4 Division of Hematology/Oncology, School of Medicine, University of Pittsburgh, Pittsburgh, PA 15213, USA

**Keywords:** deep learning, machine learning, grid search, neural networks, Bayesian networks, breast cancer metastasis, breast cancer, metastasis, prediction, EHR, clinical

## Abstract

**Background**: Unlike most cancers, breast cancer poses a persistent risk of distant recurrence—often years after initial treatment—making long-term risk stratification uniquely challenging. Current tools fall short in predicting late metastatic events, particularly for early-stage patients. **Methods**: We present an interpretable machine learning (ML) pipeline to predict distant recurrence-free survival at 5, 10, and 15 years, integrating Bayesian network-based causal feature selection, deep feed-forward neural network models (DNMs), and SHAP-based interpretation. Using electronic health record (EHR)-based clinical data from over 6000 patients, we first applied the Markov blanket and interactive risk factor learner (MBIL) to identify minimally sufficient predictor subsets. These were then used to train optimized DNM classifiers, with hyperparameters tuned via grid search and benchmarked against models from 10 traditional ML methods and models trained using all predictors. **Results**: Our best models achieved area under the curve (AUC) scores of 0.79, 0.83, and 0.89 for 5-, 10-, and 15-year predictions, respectively—substantially outperforming baselines. MBIL reduced input dimensionality by over 80% without sacrificing accuracy. Importantly, MBIL-selected features (e.g., nodal status, hormone receptor expression, tumor size) overlapped strongly with top SHAP contributors, reinforcing interpretability. Calibration plots further demonstrated close agreement between predicted probabilities and observed recurrence rates. The percentage performance improvement due to grid search ranged from 25.3% to 60%. **Conclusions**: This study demonstrates that combining causal selection, deep learning, and grid search improves prediction accuracy, transparency, and calibration for long-horizon breast cancer recurrence risk. The proposed framework is well-positioned for clinical use, especially to guide long-term follow-up and therapy decisions in early-stage patients.

## Simple Summary

Breast cancer can return even years after initial successful treatment, which makes predicting long-term recurrence very challenging. Currently available tools are not very accurate in predicting these late recurrences. In this study, we developed an advanced method using artificial intelligence to accurately predict whether breast cancer might recur at 5, 10, and 15 years after initial diagnosis. Our approach combines sophisticated techniques to identify the most relevant clinical factors, deep learning models to make precise predictions, and special methods to clearly explain how the predictions were made. By testing this method using existing medical records of breast cancer patients, we showed significantly improved prediction accuracy compared to some traditional methods. This approach can help clinicians better identify patients at high risk of recurrence and provide transparency in decision-making, potentially improving patient outcomes by guiding appropriate long-term monitoring and personalized treatment strategies.

## 1. Introduction

### 1.1. Toward Interpretable and Accurate Long-Horizon Prognostic Models

Breast cancer is one of the most commonly diagnosed cancers in women and is distinguished by its persistent risk of distant recurrence, often many years after apparently curative treatment. This long-tail recurrence risk—particularly in hormone receptor-positive subtypes—presents a clinical challenge that is less common in other cancers, where long-term survival typically implies cure. Consequently, there is a pressing need for improved prognostic tools that can guide aggressive treatment toward those likely to relapse while sparing those who are effectively cured. Although a range of molecular and tumor marker assays are in use or development for early recurrence detection, these approaches can be time-consuming, costly, and often lack validated clinical utility. An alternative strategy is to leverage large-scale clinical data and machine learning to identify histopathological and clinical predictors that enable accurate, actionable long-term risk stratification.

Despite the importance of this issue, most existing prognostic models focus on short-term endpoints (e.g., 5-year recurrence), leaving clinicians with few tools to assess long-term metastatic risk. The ability to identify patients at risk for late distant recurrence—especially among those initially diagnosed with early-stage disease—remains a critical and largely unmet clinical need.

While deep learning models have shown impressive performance in clinical risk prediction tasks, they are often criticized for their opacity and overfitting risks when trained on high-dimensional *electronic health record (HER)* data. Conversely, causal feature selection techniques such as Bayesian networks offer interpretability and parsimony but are rarely integrated with modern deep learning architectures. Few studies have systematically combined causal modeling for feature reduction with deep neural networks to build accurate and interpretable long-term prognostic tools. This methodological disconnect limits the clinical utility and generalizability of existing models.

Beyond predictive accuracy, clinical utility also demands that models be transparent and interpretable. In real-world settings, oncologists must be able to understand and trust the rationale behind a model’s prediction to inform treatment decisions. Yet most high-performing models—particularly deep learning approaches—are viewed as black boxes. Although post hoc explanation tools such as SHAP have improved model interpretability, it remains unclear whether causally selected features align with those identified as influential by SHAP. A framework that integrates causal discovery with interpretable deep learning could thus offer both high accuracy and clinical transparency, a combination that remains underexplored in the context of long-horizon breast cancer recurrence prediction.

To overcome these challenges, we introduce a novel, interpretable machine learning pipeline that combines three key innovations: (1) causal feature selection via the *Markov blanket-based interactive risk factor learner (MBIL)*, which identifies minimally sufficient feature subsets for prediction; (2) the use of *deep feed-forward neural network (DNM)* classifiers that model multivariate feature distributions without assuming linear separability; and (3) post hoc interpretation via SHAP values to enhance clinical transparency. While components such as SHAP or neural networks have been applied independently in cancer modeling, our integration of MBIL, DNM, and SHAP within a unified pipeline—focused specifically on long-term distant recurrence in early-stage breast cancer—is, to our knowledge, the first of its kind. This approach improves both predictive performance and interpretability, while addressing the unique challenge of late recurrence in breast cancer.

### 1.2. Related Work

Prior machine learning (ML) studies for breast cancer-related prediction have focused on ensemble learning, federated models, or molecular biomarker integration. For example, researchers employed ensemble frameworks or combined multi-omics and imaging features to predict short-term recurrence [1,2,3,4,5,6], but such approaches typically require high-dimensional data, often externally acquired, which may limit scalability in real-world clinical settings. In contrast, our framework uses only routinely collected clinical and histopathological data, avoiding dependence on external biomarkers.

While federated learning has been explored for privacy-preserving multi-center prediction [7], these models still face integration and harmonization challenges, and their interpretability remains limited. *Deep neural networks (DNNs)* have shown strong performance in clinical risk stratification [8,9,10,11,12], but few studies have embedded causal feature selection to constrain feature space or improve interpretability [13]. Our use of the MBIL for feature reduction and transparency—alongside SHAP-based post hoc explanation [14,15,16]—provides a novel bridge between causal reasoning and deep learning.

To our knowledge, no prior work has jointly leveraged MBIL, deep neural architectures, and SHAP to produce a long-horizon, interpretable prognostic tool for breast cancer recurrence. Furthermore, our use of grid search for optimizing DNN hyperparameters in a scalable and automated fashion supports robust generalizability, especially when working with imbalanced survival data across long time spans.

Below, we provide a background overview of the key methods of the study.

### 1.3. About Deep Learning and Grid Search

Deep learning has become an important AI-based prediction method during the last two decades [17,18,19,20]. It is a machine learning model architecture that was developed based on the *Artificial Neural Network* (*ANN*). The ANN was originally developed to recognize patterns and conduct prediction using a model structure that consists of an input layer, an output layer, and a single hidden layer of nodes, which, in a loose manner, have similar functions, such as receiving and sending out signals like neurons in the human nervous system [21,22]. Deep learning refers to a machine learning model architecture that stems from the original ANN but consists of more than one hidden layers of nodes [17,18,19,20,23,24]. Deep learning has obtained significant success in commercialized applications such as voice and pattern recognition, computer vision, and image processing [6,9,11,12,25,26,27,28,29,30,31].

**DNMs:** *Deep feed-forward neural network (DFNN) models for predicting the future risk of breast cancer metastasis* (*BCM*) can be learned from non-image clinical data concerning breast cancer [32,33,34,35]. Although the DFNN method can target other disease outcomes such as 5-year survival, we focus on BCM in this study. Therefore, we call our models *DNM* (*DFNN-BCM*). The datasets we used are two-dimensional, because they contain both columns and rows, as we see in a common two-dimensional table. In such a dataset, a column often represents an attribute or a property, for example, the stage of breast cancer, which is commonly called a variable or feature in the world of machine learning. A column contains the values of a feature from all the subjects. A row often represents a subject, for example, a patient, which is commonly called a case or data point in the world of machine learning. A row contains the values of all the features for a particular subject. We will describe the specific datasets we used in this study in the Section 2 below.

**Figure 1:**
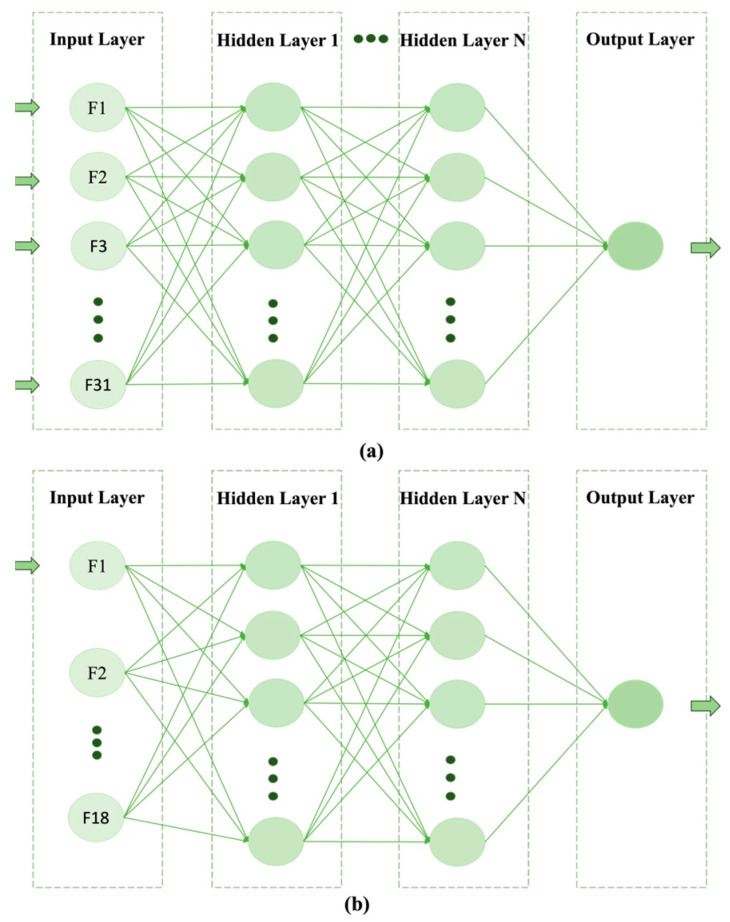
(**a**) DNM model structure; (**b**) DNM_RF model structure.

A DFNN-based model can to some extent be viewed as a “general case” of the traditional ANN model. Just like a traditional ANN model, a DFNN model contains one input layer and one output layer. But unlike a traditional ANN model that consists of only one hidden layer, a DFNN model can contain one or more than one hidden layer. Figure 1a shows the structure of our DFNN models. In these models, the input layer contains 31 nodes, representing the 31 clinical features, other than the outcome feature, contained in our datasets. The output layer contains one node, which represents our binary outcome feature called *metastasis*. *Metastasis* has two values: 0 and 1. When it is equal to 0, no metastasis is found in the patient; when it is equal to 1, metastasis is found in the patient.

**Grid search:** The prediction performance of a DFNN-based model that is learned from data is closely associated with a learning scheme called grid search [34,35]. There are a large number of adjustable hyperparameters in a deep learning method like the DFNN, and different value assignments for the set of adjustable hyperparameters can result in models that perform differently. This can be considered an advantage of deep learning because more adjustable hyperparameters allow us to have more ways of changing and improving a model. But on the other hand, having a large number of adjustable hyperparameters makes it a more challenging task to conduct hyperparameter tuning, which is the process of determining an optimal value assignment for all hyperparameters. Grid search can be considered a systematic way of conducting hyperparameter tuning [34,36,37]. We describe the procedure of our grid searches as follows: Firstly, we determine a set of values for each of the adjustable hyperparameters. For example, an adjustable hyperparameter called *learning rate* can technically take an infinite number of different values ranging from 0 to 1; therefore, we need to select a fixed number of values for *learning rate*. Secondly, we give the preselected sets of values for the adjustable hyperparameters to our grid search program as one of its inputs. Thirdly, we run our grid search program, which conducts an independent model training and testing process at every unique value assignment of the set of hyperparameters, determined by the sets of input hyperparameter values. Such a unique value assignment of the set of adjustable hyperparameters is called *hyperparameter setting* (*HYPES*) in our research [34,35]. Finally, our grid search program automatically stores as one of its outputs the HYPES and corresponding model performance scores resulting from each of the independent model training and testing processes.

### 1.4. About Bayesian Networks

**Figure 2:**
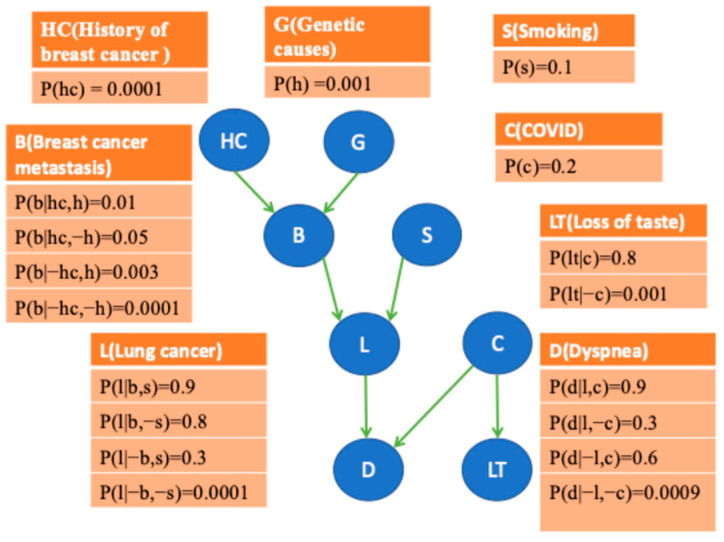
A hypothetical medical BN.

**Bayesian networks:** *Bayesian networks* (*BNs*) have become a leading architecture for modeling uncertain reasoning in artificial intelligence and machine learning [38,39,40,41,42]. A Bayesian network consists of a *directed acyclic graph (DAG)*, whose nodes represent random variables, and the conditional probability distribution of every variable in the network given each set of values of its parents [42]. We will use the terms node and variable interchangeably in this context. Directed edges represent direct probabilistic dependencies. In general, for each node *X_i_*, there is a probability distribution on that node given the state of its parents, which are the nodes with edges going into *X_i_*. The nodes that can be reached by following a directed path from *X_i_* (following a tail-to-head direction of the edges) are called the descendants of *X_i_*. For example, in the eight-node hypothetical medical BN shown in Figure 2, node *D* has two parents, which are *L* and *C*, and node *S* has two descendants, which are *L* and *D*. A BN encodes a joint probability distribution, and therefore, it represents all the information needed to compute any marginal or conditional probability on the nodes in the network. A variety of algorithms have been developed for rapidly computing P(XS1|XS2), where XS1 and XS2 are arbitrary sets of variables with instantiated values [39,42,43].

**Figure 3:**
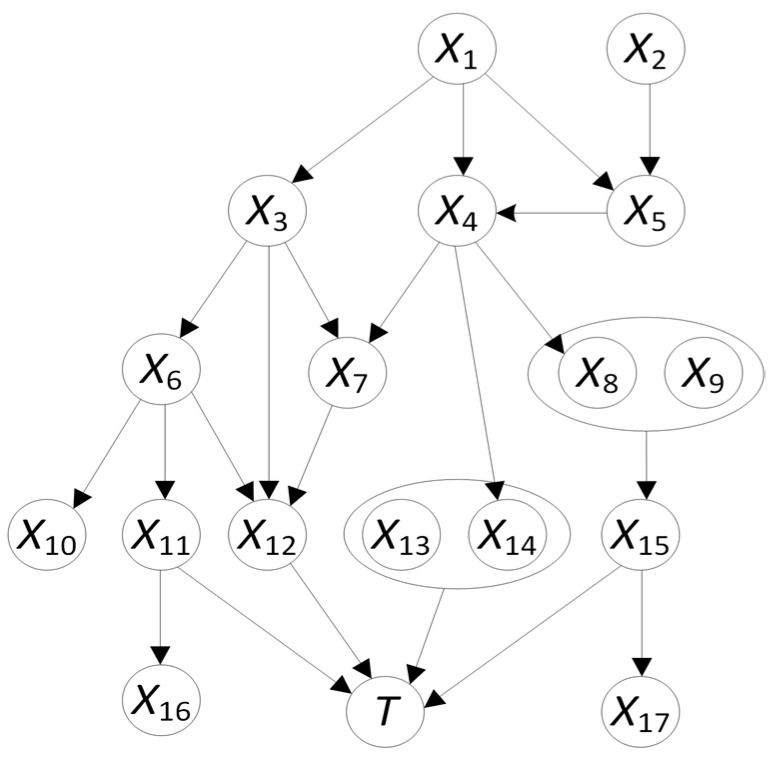
A BN model demonstrating the dependency and independency relationships among variables [33]. The Markov blanket of *T* consists of nodes *X*_11_, *X*_12_, *X*_13_, *X*_14_, and *X*_15_. These nodes separate *T* from the noisy predictors *X*_1_–*X*_10_, *X*_16_, and *X*_17_.

**Markov Blanket and MBIL:** In a BN, a *Markov blanket* of a given node *T* contains at least the set of nodes *M* such that *T* is probabilistically independent of all other nodes in the network conditional on the nodes in *M* [42]. In general, a Markov blanket of *T* contains at least all parents of *T*, children of *T*, and parents of children of *T*. If *T* is a leaf (a node with no children), then a Markov blanket consists only of the parents of *T*. Figure 3 shows a BN DAG model. Since *T* is a leaf in that BN, a Markov blanket *M* of *T* consists of its parents, namely nodes *X*_11_–*X*_15_. Without knowing the BN DAG model, nodes *X*_1_–*X*_10_, *X*_16_, and *X*_17_ would all be learned as predictors because they are indirectly connected to *T* through the nodes in the Markov blanket *M* [33]. However, if we can identify *M* and know the values of the nodes in it, we will have blocked the connections between *T* and the other nodes. So, the other nodes can be completely removed from our prediction model without affecting the prediction performance of the model. This helps reduce the complexity of a prediction model, and therefore could hypothetically improve prediction performance and reduce computational cost, which is one of the challenges for deep learning with grid search [34,36,37,44]. Based on this idea, we previously developed the *Markov blanket-based interactive and direct risk factor learner* (*MBIL*) [33], a supervised BN-based method for learning causal risk factors for a target feature such as BCM. We developed MBIL based on the Bayesian network learning techniques and its relevant concepts such as Markov blankets [33,42], as described above. MBIL can be used for identifying risk factors for a target feature [33]. It detects not only single causative risk factors in a Markov blanket of a leaf target node but also interactive factors that jointly affect such a target node [33].

While model performance is essential, clinical deployment also demands interpretability. Black-box models like deep neural networks often lack transparency, limiting clinician trust and routine adoption. Conversely, interpretable models tend to sacrifice predictive accuracy. Although post hoc explanation methods such as SHAP have emerged to bridge this divide, few studies have explicitly assessed whether features selected by causal discovery methods also align with SHAP-based importance rankings. This unexplored synergy between causally relevant features and interpretable deep learning remains a gap in long-term cancer risk modeling.

### 1.5. About the Purpose of This Study

In this study, we developed and optimized both DNM and *DNM_RF* models through well-designed grid searches. RF stands for risk factor. We developed DNM_RF models by applying the MBIL package (v0.0.1), which can be used to learn interactive and direct risk factors [33]. We first learn RFs that are predictive of BCM from our datasets, which contain 31 clinical features (see details in Table S1 of the Supplementary Materials). Then, we retrieve new datasets using the RFs. The DNM_RF models are learned from the new datasets. Therefore, the input layer of the DNM_RF models contains a smaller number of features than that of the DNMs, as demonstrated in Figure 1. We describe in detail how the DNM and DNM_RF models are developed in the Section 2 below.

The main purpose of this study is to compare the DNMs and the DNM_RF models. We also want to compare the DFNN-based models with some other typical machines learning (*ML*) models, which are all developed through grid searches. The set of ML methods we used in this study are described in the Section 2. We made the following assumptions: (1) *a DNM_RF prediction model will yield better predictive performance in grid search than the corresponding DNM prediction model and* (2) *a DFNN-based prediction model will yield better predictive performance than the representative set of other ML-based prediction models.* We made these assumptions because (1) using the RFs that are found by the BN-based MBIL to guide prediction will help reduce model complexity and tease out the “noises” made by the non-BCM-predictive features, and this could lead to a better prediction performance and reduce time for grid search; (2) DFNN has a large set of adjustable hyperparameters, which allows sophisticated hyperparameter tuning to improve prediction and reduce overfitting; and (3) deep learning is a popular and powerful prediction tool, as demonstrated by its successes in many other applications.

## 2. Methods

### 2.1. Datasets

We used six datasets concerning BCM in this study. Among them, LSM-5Year, LSM-10Year, and LSM-15Year were developed and made available through previous studies [32,33]. During these previous studies, we eliminated all variables that were redundant or composites of other variables (e.g., *stage*) and all variables that have 1/3 or more missing values. So, the candidate risk factors included in this study are the ones shown in Table S1. We assigned the value *yes* to metastasis if the patient metastasized within 5 years of initial diagnosis, the value *no* to metastasis if it was known that the patient did not metastasize within 5 years, and the value NULL to metastasis if the patient discontinued follow-up within the first five years and had not metastasized. The value NULL was also assigned to all missing data fields in all variables. Missing data (NULL values) were filled in using the *nearest neighbor* imputation algorithm.

The LSM_RF-5Year, LSM_RF-10Year, and LSM_RF-15Year were developed using the RFs identified by the MBIL method [33]. Using the LSM_RF-5Year as an example, the 2-step procedure for curating this dataset is as follows: Step 1: Applying the MBIL method to the LSM-5Year to retrieve the RFs that are predictive of BCM. The LSM-5Year contains 32 variables including an outcome variable called *metastasis*, which represents the state of having or not having BCM by the 5th year post the initial treatment. Sometimes we call the outcome variable the target feature. The remaining 31 variables are the predictive features, which are also referred to as predictors when they are used to predict a patient outcome. Step 2: Removing from LSM-5Year all columns of the non-outcome features that do not belong to the set of RFs found in Step 1, with the remaining part of the data forming LSM_RF-5Year. We followed the same 2-step procedure to obtain LSM_RF-10Year from LSM-10Year and LSM_RF-15 year from LSM-15Year. Table 1 below shows the counts of the cases and predictors included in the six datasets. More detailed descriptions of the predictors for the six datasets are included in Tables S1–S4 of the Supplementary Materials.

### 2.2. The RGS Strategy and the RGSP for Developing DNM and DNM_RF Models

We developed *the Randomized Grid Search Package (RGSP)* python package (v0.0.1), which can be used to develop and optimize the DFNN types of models. RGSP contains the following major components other than the routine dataset processor and output generator: (1) The DFNN model builder, which uses the libraries provided by the Keras package (v2.4.3) [45]. Keras is a high-level neural network API built on top of TensorFlow [45,46]. The Keras package is made available in a collection of python packages named Scikit-Learn (v0.24.2) [45]. TensorFlow is an open source development platform for machine learning and artificial intelligence (AI), and Keras can be viewed as a wrapper of TensorFlow. Such a wrapper serves as a communication interface between a deep learning developer and TensorFlow. (2) The DFNN model learner, which follows the k-fold *cross validation* (*CV*) strategy to be used to train and test DFNN models by calling the DFNN model builder. Since k-fold CV is also closely related to the evaluation of DFNN models, more detailed information about this component is seen in the “Performance Metrics” subsection (Section 2.6) below. (3) The *Randomized Grid Search* (*RGS*) *Hyperparameter Setting Generator* (*RGS_HSG*), which takes as input a preselected set of values for each of the adjustable hyperparameters to produce randomly selected HYPESs. We call all possible HYPESs that are determined by the input sets of hyperparameter values the *pool of hyperparameter settings* (*P-HYPESs*). The number of HYPESs in the pool can be very large. Using the sets of hyperparameter values we used in our experiments concerning DFNN method (as shown in Table S5 of the Supplementary Materials) as an example, the number of available unique HYPESs in the correspond P-HYPESs is the product of 332, 4189, 299, 90, 89, 299, 299, 299, 4, 4189, and 400. So, running a “full” grid search, that is, using every HYPES in a corresponding P-HYPESs to train and test models, is often not feasible. The key point of our RGS strategy is to randomly generate a certain number of HYPESs from the corresponding P-HYPESs following a uniform distribution, so that the grid search can be finished within a reasonable timeframe and every HYPES in the pool has an equal chance to be picked. RGS_HSG implements this strategy. (4) A grid searcher, which was developed based on the grid search libraries provided by Scikit_Learn. The grid searcher goes through every HYPES generated by RGS_HSG, and at each HYPES, it calls the DFNN model learner to train and test models using the current HYPES and records the output information such as the current HYPES and the corresponding model performance scores.

In this study, we learned DNMs from the LSM datasets using RSGP. Specifically, we learned the DNM-5Year models from the LSM-5Year dataset, the DNM-10Year models from the LSM-10Year dataset, and the DNM-15Year models from the LSM-15Year dataset. These models are the all-feature models. Similarly, we learned the DNM_RF-5Year models from the LSM_RF-5Year dataset, the DNM_RF-10Year models from the LSM_RF-10Year dataset, and the DNM_RF-15Year models from the LSM_RF-15Year dataset, and these models are the RF models. We describe in detail the grid search experiments we conducted to develop these models in the “Experiments” subsection (Section 2.5) below. We can predict for a new patient the risk of 5-year BCM using the DNM-5Year and DNM_RF-5Year models, 10-year BCM using the DNM-10Year and DNM_RF-10Year models, and 15-year BCM using the DNM-15Year and DNM_RF-15Year models.

### 2.3. The Extended RGSP for Developing the Comparison ML Models

As stated in the Introduction, another main purpose of this study is to compare the DFNN-based models with a set of representative ML models that are not based on ANNs. We included the following ML methods in this study: *Naïve Bayes* (*NB*), a simplified Bayesian network (BN) model that normally only contains one parent node and a set of children leaf nodes [42,47,48]. In a basic NB model, there is an edge from the parent to each of the children. There are multiple types of NB classifiers included in the Scikit-Learn libraries. We used the categorical NB in this study because our datasets only contain categorical data. *Logistic Regression* (*LR*), a supervised learning classification method that is normally suitable for binary classification problems [48,49]. We included this method because our outcome feature is a binary variable. *Decision tree* (*DT*), one of the most widely used machine learning methods. It contains a tree-like structure in which each internal node represents a test on a feature and each leaf node represents a class value [50]. It can be used for both classification and regression tasks. *Support Vector Machine* (*SVM*), a machine learning method that tries to identify a hyperplane that has the maximum margin defined by support vectors [51,52]. SVM can be used for both regression and classification tasks, and it is widely applied in the latter. We used the SVC version of the SVM in this study, which uses a linear hyperplane to separate the data points. We therefore use SVM and SVC as exchangeable terms in this paper. *The least absolute shrinkage and selection operator* (*LASSO*), a regression-based classifier that can be used to conduct variable selection and regularization in order to enhance prediction performance [53]. *K-Nearest Neighbors* (*KNN*), a supervised machine learning method that can be used for both classification and regression tasks [54]. KNN predicts the class value of a new case with its *k* nearest neighboring data points. To do this, KNN assumes that cases with similar covariate values are near to each other [54]. *Random forests* (*RaF*), a typical bagging type of ensemble method, in which the trainer will randomly select a certain amount of sample data and create corresponding decision trees to form a random forest [55]. *Adaptive Boosting* (*ADB*), a typical boosting type of ensemble method. Unlike the RaF model, where each DT is independent, the next learner of ADB will adjust its prediction work based on the result of the previous weak learner that tends to make incorrect predictions [56]. *eXtreme Gradient Boosting* (*XGB*), another well-known boosting type of ensemble learning. Unlike ADB, it uses gradient boosting, which is based on the difference between true and predicted values to improve model performance [57].

We used the libraries provided in Scikit-Learn [45,58] to implement these ML classifiers. Just like the deep learning method, each of these ML methods has a set of adjustable hyperparameters (see Table S5 in the Supplementary Materials) that can be tuned to optimize prediction performance. We extended our RGSP to include the nine ML methods. As we did for the DFNN method, we conducted grid searches using RGSP to learn and optimize the all-feature models from the LSM datasets, and the RF models from the LSM_RF datasets for each of the nine ML methods. For the nine ML methods, the all-feature models are named with their short method names, and the RF models are named as the short method names concatenated with ‘RF’. For example, the all-feature models for the *Naïve Bayes* method are called NB models, and the RF models for the *Naïve Bayes* method are called NB_RF models.

### 2.4. The Adjustable Hyperparameters and Their Value Selection

As previously described, the RGS_HSG component of RGSP takes as an input a preselected set of values for each of the adjustable hyperparameters and produces for a grid search a certain number of HYPESs randomly selected from the P-HYPESs. The number of HYPES is another input parameter of RGS_HSG. The P-HYPESs are determined by the sets of input values, one for each of the adjustable hyperparameters. The general rules we used to select an input set of values for an adjustable hyperparameter are as follows: if a hyperparameter contains a fixed number of values, then give all of them to RGS_HSG; if a hyperparameter has an infinite number of values, for example, when it is a continuous variable, we first identify the minimum and the maximum values of this hyperparameter using a package called *Single Hyperparameter Grid Searches* (*SHGS*) (v1.0.2) [59]. We then select all values between the minimum and maximum values, separated by a multiple of a very small value called step size. For example, if for a hyperparameter, the minimum value is 0.001 and the maximum value is 0.3, and if we use a step size of 0.001, then the set of values that we choose for the hyperparameter would include a sequence of 299 different values, which are 0.001, 0.002, 0.003, …, 0.298, 0.299, and 0.3. We applied these rules to all ten ML methods involved in this study including deep learning. The adjustable hyperparameters and their input values that we used for the ten ML methods are shown in Table S5 in the Supplementary Materials.

### 2.5. Experiments

To compare the RF models with corresponding all-feature models for each of the ten ML methods including deep learning, we discovered the best RF and all-feature model for predicting 5-year, 10-year, and 15-year BCM each. This gives us the 6 best models per method, and 60 best models in total for the 10 methods. We followed the same experiment procedure to identify each pair of the best RF and all-feature models, which we describe below, using the development of the best DNM-5Year and DNM_RF-5Year models as an example.

*Step 1*. Call RGS-HSG to randomly generate 6000 HYPESs. The input set of values for each of the hyperparameters to RGS-HSG is shown in S5, and the input number of HYPESs to RGS-HSG is 6000. *Step 2.* Run the grid searcher of RGSP. The set of 6000 HYPESs generated in Step 1 is one of the inputs to the grid searcher. Another input to the grid searcher is the dataset, namely, the 80% of the LSM-5Year data that serves as the training–test set (see “performance metrics”). The grid searcher will go through each of the 6000 HYPESs and train and test models following the 5-fold CV mechanism (see “performance metrics”) at each HYPES. At each of the 6000 settings, five different models are trained and tested, and performance scores regarding model training and testing and the corresponding HYPES are all recorded as part of the output of the grid searcher. During this step, 30,000 DNM-5Year models are trained and tested. *Step 3.* At the end of the grid search, the grid searcher will select the best HYPES that is associated with the best average performance score among all 6000 HYPES. The top DNM-5Year model will then be developed by refitting the entire training–test set of the LSM-5Year data using the best HYEPS. *Step 4.* Repeat Step 2 and Step 3 to develop the best DNM_RF-5Year model, but using the LSM_RF-5Year dataset instead of the LSM-5Year dataset.

In order to ensure a fair comparison between a pair of best RF and all-feature models, we used the exact same 6000 HYPESs generated in the same Step 1, while followed separate Steps 2 and 3 when developing the two best models. But a different Step 1 was conducted for each different dataset and ML method (see Figure S12). To ensure a fair and unbiased comparison of computational efficiency across methods, we performed separate grid search optimizations for each model under standardized computational conditions. Specifically, we executed all grid search experiments using the same pool of machines, with each job allocated an equal number of CPU cores and RAM (16 cores and 64 GB RAM per run). No GPU was used, and early stopping was not used. The workload was manually distributed across a small, dedicated cluster to ensure balanced utilization and reproducibility. This controlled setup minimizes potential variability due to resource contention or hardware differences, thereby supporting the validity of the reported timing and performance comparisons.

### 2.6. Performance Metrics and 5-Fold CV

**ROC curve and AUC**: Our grid searches use an *area under the curve (AUC)* score to measure the prediction performance of a model. AUC originated from what is called a *receiver operator characteristic curve* (*ROC curve*), which plots the *true positive rate* (*TPR*) against the *false positive rate* (*FPR*) at each of the cutoff values, given a test dataset and a prediction model [60]. The TPRs and FPRs are calculated based on the set of true outcome values contained in the test dataset and the corresponding set of predicted outcome values obtained from the prediction model. An AUC score measures the discrimination performance of a model.

**The 5-fold CV procedure, mean-test AUC, and validation AUC**: Our grid searchers follow the 5-fold CV to train and test models at each HYPES. In order to conduct a 5-fold CV in a grid search, we first split the dataset that will be used in the grid search. In general, we used the following procedure to split a dataset: (1) Split the entire dataset into a train-test set that contains 80% of the cases and a validation set that contains 20% of the cases. The training–test set was given to a grid search as the input dataset, and the validation set was kept aside for later validation tests. (2) Divide a training–test set evenly into 5 portions for the purpose of conducting 5-fold CV. The division was mostly performed randomly except that each portion should have approximately 20% of the positive cases and the negative cases, respectively, to ensure that it is a representative fraction of the dataset. During a 5-fold CV, five different models are generated and tested, and each is trained using a unique combination of four portions of the training–test set and tested using the remaining portion. Five AUC scores are produced based on the tests undertaken by the five models, and the average of these scores, called the mean-test AUC, is also computed and recorded. The best HYPES is selected at the end of a grid search is based on the mean-test AUC scores recorded, and the best model is developed by refitting the entire training–test set used by the grid search at the best HYPES. In this study, a ROC curve for a selected prediction model was generated by testing the cases contained in the corresponding validation set using the model, and we call the AUC obtained from such a curve a validation AUC.

### 2.7. The SHAP Method for the Explanation of a Prediction

The Shapley value was introduced by Lloyd Shapley in 1951. It represents the distribution of individual payoffs in cooperative games by measuring the marginal contribution of an individual to the collective outcome [15,16]. The Shapley additive explanations (SHAP), developed based on the Shapley value, is a method that can be used to explain the predictive output of a machine learning model [14,61]. A SHAP value shows the importance of a feature for contributing to the predicted outcome value. How a SHAP value is computed can be explained using the following formula [14]:

φip=1F∑S⊆F\{i}[p(S∪i)−pS]F−1S

Each additive term of this formula has two components: (1) the marginal contribution of the *i*th feature to the model’s prediction; (2) the weight associated with the marginal contribution. *F* represents the complete set of all features contained in the data, *i* means the *i*th feature, for which we are computing the SHAP value, and *S* represents a subset of *F*, which excludes the *i*th feature. p(S∪{i}) represents the model’s predicted outcome value using the combined set of features in S and {*i*} as the predictors, while p(S) represents the model’s predicted outcome value using only the features in S as predictors. p(S∪{i}) − p(S) represents the contribution to the model’s prediction, made by adding the *i*th feature to the subset S. For each subset, 1/(FF−1S) is given as the weight, determined by |*F*|, the size of the complete set of features F, and |*S*|, the size of S. The purpose of using the weight is to balance the overall prior influence among all possible sizes of S.

In this study, we used the Kernel Explainer of the SHAP library [14] to conduct SHAP analyses concerning the 60 best prediction models we obtained for the ten ML methods. To compute SHAP values, we first identify *k* representative cluster centroids from a training–test set using the *k*-means clustering method. We then obtained the *background values* of features by computing the mean of the corresponding feature values of the *k* centroids. In order to compute the SHAP value for the *i*th feature, the Kernel Explainer generates synthetic samples and used them as the test cases for a model [14]. Each of the synthetic samples contains the true values from a validation set for a subset *S* together with the background values for the remaining features in *F* except for the *i*th feature. The *i*th feature assumes its true value from the validation set in a synthetic sample when the sample is used to obtain the p(S∪{i}) from the model and assumes its corresponding background value in a synthetic sample when it is used to obtain the p(S) from the model. We generated SHAP bar plots, which show the mean absolute SHAP values of features from all test cases, and summary plots, which show the SHAP value distributions of features among all test cases.

## 3. Results

Table 2 below shows the side-by-side comparison of the best DNM and DNM_RF models in terms of their prediction performance measured by the mean-test AUC for predicting 5-year, 10-year, and 15-year BCMs, respectively. We extended our experiments by conducting the same comparison for each of the nine non-deep learning ML methods, and the results are also included in Table 2. In addition, Table 2 contains a column named “% Difference”, which shows the percentage increase or decrease in the mean-test AUC of a best RF model from the corresponding best all-feature model. For example, for DNMs that predict 5-year BCM, the percentage difference is −2.09%, which means that the best DNM_RF-5Year model performs worse than the DNM-5Year model by 2.09% in terms of mean-test AUC.

As previously described in the Experiments section (Section 2.5), each of the 60 best models that we obtained was developed based on the best HYPES that was selected from 6000 randomly picked HYPESs used in corresponding grid searches, based on the mean-test AUC of the five models trained at each HYPES. In addition to developing and comparing the best models obtained through grid searches, we are also interested in knowing and comparing the average performance of all models trained during corresponding grid searches. Due to this, we created Table 3 below, which contains, for each of the 60 best models, the average value of the 6000 mean-test AUCs associated with the 6000 HYPESs used in corresponding grid searches.

Each of the best models was obtained at the cost of training and testing tens of thousands of models through grid searches. A grid search with deep learning can be very time consuming. Table 4 below summarizes the running time used by our grid searchers. We arranged these results in a side-by-side manner to compare the grid search time used for developing the best RF model with that used for developing the corresponding best all-feature model.

**Figure 4:**
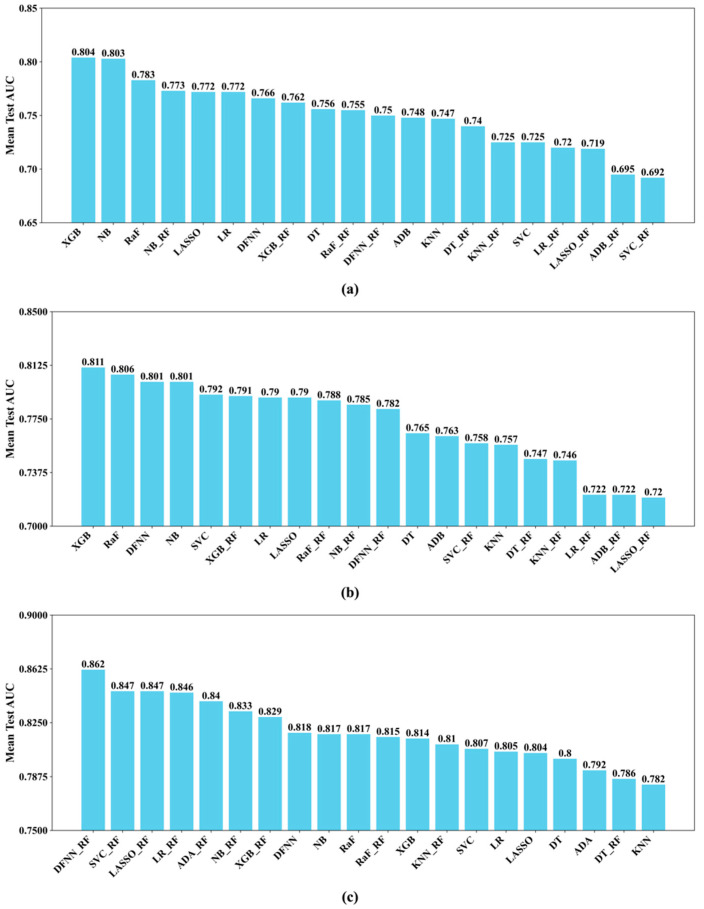
The rankings of the 20 best ML models shown in a bar chart: (**a**) 5-year; (**b**) 10-year; (**c**) 15-year.

Another purpose of this study is to compare the prediction performance of the ten ML methods including deep learning. For predicting 5-year BCM, we compare in Figure 4a below the 20 best prediction models that we developed for the ten ML methods, including 10 best all-feature models and 10 best RF models. We also rank the mean-test AUC scores of the 20 models from high to low and show the rankings using a bar chart in Figure 4a. We did the same for the best 20 models for predicting 10-year BCM in Figure 4b and for predicting 15-year BCM in Figure 4c.

**Figure 5:**
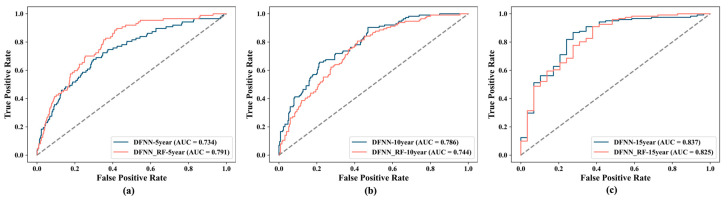
Side-by-side comparison of the ROC curves for the best DNM and DNM_RF model concerning predicting 5-year (**a**), 10-year (**b**), and 15-year (**c**) BCM.

**Figure 6:**
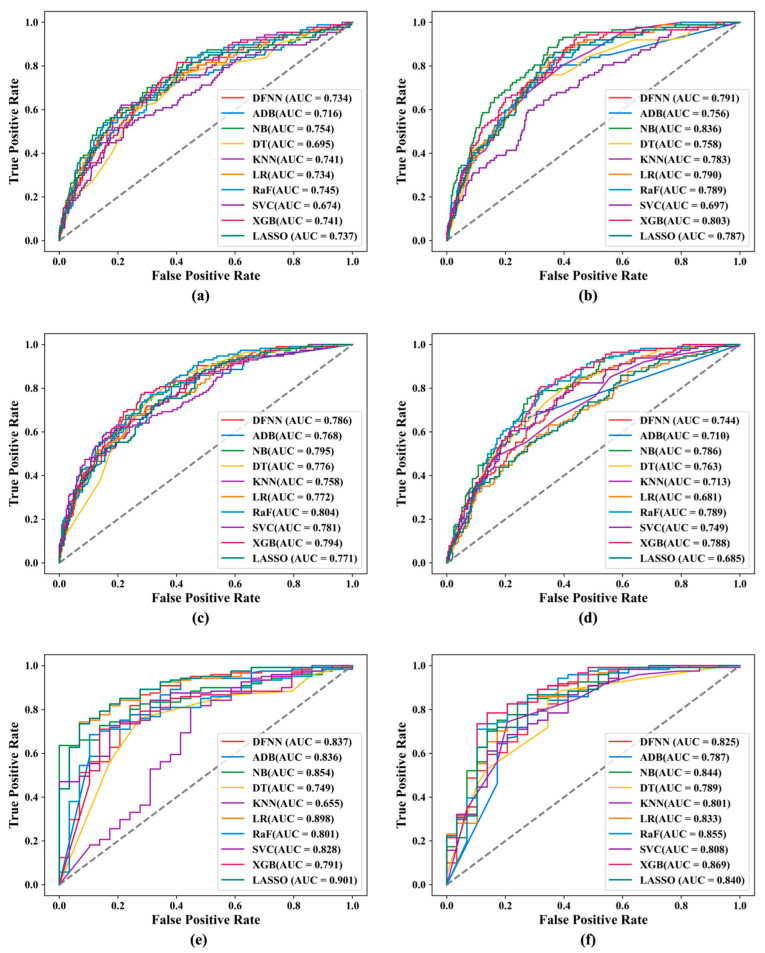
ROC curves for the best models of the ten ML methods: (**a**) all-feature 5-year; (**b**) RF 5-year; (**c**) all-feature 10-year; (**d**) RF 10-year; (**e**) all-feature 15-year; (**f**) RF 15-year.

We also developed a ROC curve for each of the 60 best models. Such a curve was developed by plotting FPRs against TPRs, obtained by testing cases in an independent validation set using one of the best models (see Section 2 for details). In Figure 5 below, we compare, side by side, the ROC curves created for the best DNM and DNM_RF model concerning the risk prediction of 5-year (Figure 5a), 10-year (Figure 5b), and 15-year (Figure 5c) BCM, respectively. Figure 6 compares the prediction performance of the ten ML methods in terms of their corresponding best models. It contains a panel of six subfigures. Each subfigure consists of 10 ROC curves created for the corresponding best models of the ten ML methods. For example, Figure 6a contains the 10 curves for the best all-feature ML models concerning 5-year BCM.

Our results indicate favorable Brier scores across different time horizons, reflecting well-calibrated probabilistic outputs (Figures S13–S15). For the best 5-year model (XGB_5Year), we obtained a Brier score of 0.085, which falls within the range typically considered excellent in clinical prediction settings. The best 10-year model (XGB_10Year) yielded a Brier score of 0.164, and the best 15-year model (DNM_RF_15Year) achieved a Brier score of 0.107. These scores support the reliability of our models’ risk estimates over both medium- and long-term intervals, which is essential for clinical decision-making in the context of breast cancer recurrence.

**Figure 7:**
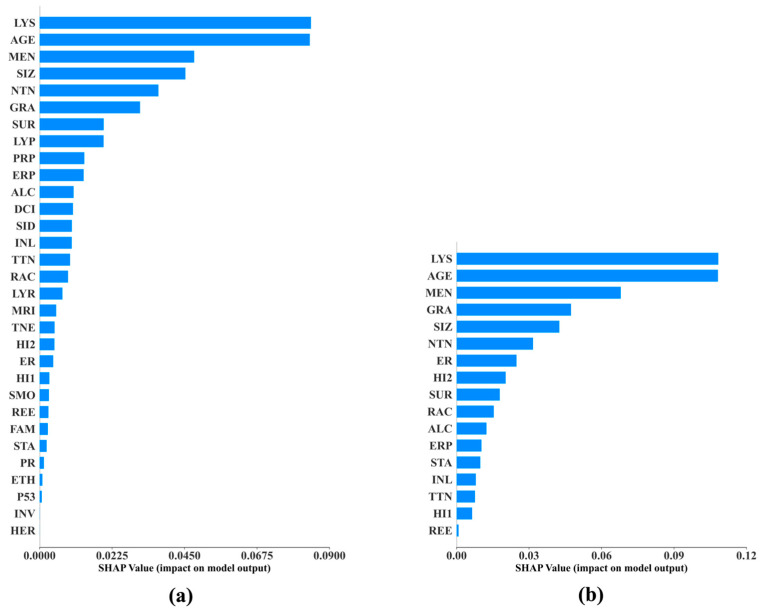
The SHAP bar plots for the best DNM_15-Year model (**a**) and DNM_RF-15Year model (**b**).

**Figure 8:**
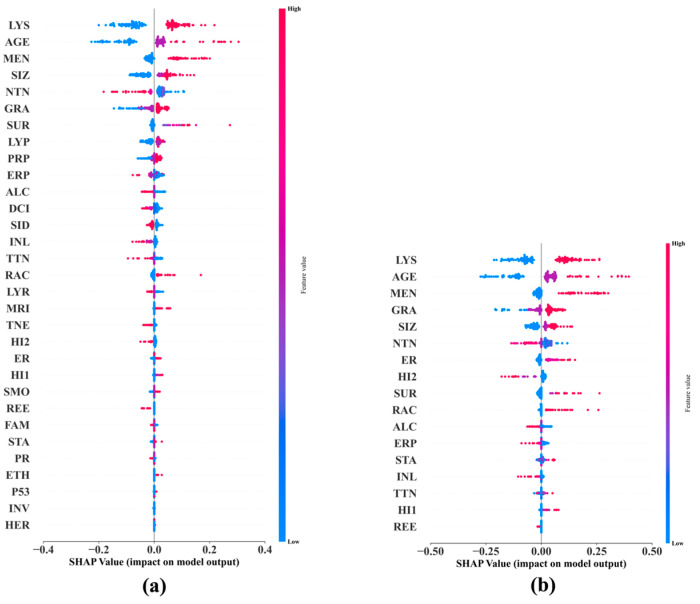
The SHAP summary plots for the best DNM-15Year model (**a**) and DNM_RF-15Year mode (**b**).

We conducted SHAP analyses and developed a SHAP feature importance plot and summary plot for each of the 60 best prediction models. Due to the page limit, we do not include all of the 120 plots in this paper; instead, we show, side by side, the SHAP bar charts of the two best DNMs concerning 15-year BCM in Figure 7 and the SHAP summary plots of these two models in Figure 8, as an example. We include in the Supplementary Materials the SHAP summary plots for the two best DNMs concerning 5-year BCM and 10-Year BCM, respectively, and the two best models of each of the top three ML methods excluding DNM concerning 5-year, 10-year, and 15-year BCM, respectively. This gives us 22 plots included in 11 figures, namely, Figures S1–S11 in the Supplementary Materials. The top ML methods were selected based on the rankings shown in Figure 4.

### Comparison of SHAP and MBIL in Feature Interpretation

To assess the consistency between model-intrinsic feature importance (via SHAP) and risk factor selection (via MBIL), we compared the top predictors identified by both methods across the best-performing models at each prediction horizon (5, 10, and 15 years). For late-stage predictions (15-year BCM), SHAP and MBIL generally converged on key factors such as lymph node involvement, tumor size, and hormone receptor status (e.g., ER/PR). However, in the 5-year and 10-year models, partial discrepancies emerged.

Specifically, MBIL tended to identify features with direct probabilistic causality, such as TNEG (triple-negative status) or histology subtype, whereas SHAP highlighted features like patient age and menopausal status with strong marginal contributions in the trained model. This difference reflects their distinct conceptual roles: MBIL focuses on discovering Markov blankets and causally informative subsets prior to model training, whereas SHAP measures post hoc impact within a fixed model structure.

We observed strong convergence between the features selected by MBIL and those identified as influential by SHAP value analysis across all prediction horizons. As shown in Supplementary Materials Figure S8, for the 15-year outcome, nearly all features prioritized by MBIL—such as nodal status, tumor size, and hormone receptor expression—also ranked among the top SHAP contributors. Even at shorter horizons, while SHAP occasionally emphasized additional features such as age and tumor grade, MBIL-selected variables continued to exhibit above-average SHAP values. The MBIL feature subsets listed in Table 2 confirm this overlap across models. These findings demonstrate that MBIL is effective not only in identifying compact, causally informative feature sets but also in preserving clinically meaningful variables that remain impactful within model predictions. This alignment reinforces the interpretability and trustworthiness of our modeling pipeline, supporting the dual utility of MBIL in both dimensionality reduction and clinical insight.

Acronyms for Figure 7 and Figure 8: ***AGE***: *age at diagnosis of the disease*; ***ALC***: *alcohol usage*; ***DCI***: *type of ductal carcinoma* in situ; ***ER***: *estrogen receptor expression*; ***ERP***: *percent of cell stain pos for ER receptors*; ***ETH***: *ethnicity*; ***FAM***: *family history of cancer*; ***GRA***: *grade of disease*; ***HER***: *HER2 expression*; ***HI1***: *tumor histology*; ***HI2***: *tumor histology subtypes*; ***INL***: *where the invasive tumor is located*; ***INV***: *whether tumor is invasive*; ***LYP***: *number of positive lymph nodes*; ***LYR***: *number of lymph nodes removed*; ***LYS***: *patient had any positive lymph nodes*; ***MEN***: *inferred menopausal status*; ***MRI***: *MRIs within 60 days of surgery*; ***NTN***: *number of nearby cancerous lymph nodes*; ***PR***: *progesterone receptor expression*; ***PRP***: *percent of cell stain pos for PR receptors*; ***P53***: *whether P53 is mutated*; ***RAC***: *race*; ***REE***: *removal of an additional margin of tissue*; ***SID***: *side of tumor*; ***SIZ***: *size of tumor in mm*; ***SMO***: *smoking*; ***STA***: *composite of size and # of positive nodes*; ***SUR***: *whether there is residual tumor*; ***TNE***: *triple negative status in terms of patient being ER-*, *PR-*, *and HER2-negative*; ***TTN***: *prime tumor stage in TNM system*.

## 4. Discussion

One of the main purposes of this study is to compare the DNM_RF models with the DNMs, and we assumed that the DNM_RF models should perform better. Our experiments demonstrate that this is indeed true in certain situations. Specifically, as shown in Table 2, when predicting 15-year BCM, the DNM_RF-15Year model (with a mean-test AUC of 0.862) performs about 5.4% better than the DNM_15Year model (with a mean-test AUC of 0.818). In addition, according to Figure 5a, when predicting 5-year BCM, the DNM_RF-5Year model (with a validation AUC of 0.792) performs almost 8% better than the DNM-5Year model (with a validation AUC of 0.734). As described in the *Performance Metrics* subsection (Section 2.6), a validation AUC is obtained by making predictions for the cases in the 20% data that were saved prior to grid searches; that is, these cases do not participate in any model training process. Therefore, a validation AUC reflects a model’s capability of making correct predictions for new patients it has never seen. Thus, Figure 5a reveals that the DNM_ RF-5Year model is 8% more capable than the DNM-5Year model when dealing with new patients. Finally, if we consider all of the 60 best prediction models we obtained for the ten ML methods including deep learning, DNM_RF-15Year is the best of the best by producing the highest mean-test AUC we saw in our study. As shown by the bar chart in Figure 4c, when predicting the risk of 15-year BCM, the DNM_RF model performs significantly better than all other best ML models including the best DNM model.

Recall that the DNM_RF-15Year model uses only a fraction of the features used by the DNM-15Year model, but it beats the latter when predicting the risk of 15-year BCM. This demonstrates the power of a BN-based method like MBIL in fishing for risk factors that are critical to a prediction. In addition, the results reveal the potential strength of a collaborative effort from different AI-based approaches such as deep learning and Bayesian networks. As we know, DNM_RF-15Year, which scored the highest among all of the best models, is a model obtained from a coalition of deep learning, a Bayesian network, and grid search. The DNM_RF-5Year model that outperforms DNM-5Year by 8% when making predictions for new patients is also a result of such a collaborative effort.

Although an RF model outperforms its corresponding non-RF model in eight out of the ten ML methods when predicting 15-year BCM, as demonstrated by Table 2 and Figure 4, similar results are not seen for models that predict the risks of 5-year and 10-year BCM. We reckon this may somewhat further indicate the power of combing an ML method with grid search, since an all-feature model is a result of the coalition of these two. Recall we assumed that an RF model should beat its corresponding all-feature model mainly because the nonpredictive features remaining in the all-feature model may become noisy and therefore hinder its prediction capability. The results regarding 5-year and 10-year BCM may just tell us that the expected “noisy” effects have never occurred or were offset by something else, such as the coalition with a grid search. Without the “noisy” effects, a good all-feature model should indeed be at least no worse than the RF one, as seen in the 5-year and 10-year cases, because all the good predictors in the latter are also available in the former, while some weak predictors that can be overlooked by a risk factor learner such as MBIL would only be available in the all-feature model.

The average mean-test AUC of all corresponding models trained, as seen in Table 3, shows the expected model prediction performance when the HYPESs of a grid search are randomly picked from the P-HYPESs. This should also be the expected model performance when the HYPES of a model is randomly selected from the P-HYPES without performing a grid search. Based on Table 3, five out of the ten ML methods, including XGBoost, DFNN, RaF, DT, and SVM, benefit greatly from grid searches, with a percentage performance increase from that of the best model discovered through grid searches to the expected performance at random without grid searches, ranging from 25.3% (with SVC_RF-15Year) up to 60% (with XGB_RF-15Year). Using the DNM_RF-15Year models as an example, the average mean-test AUC of all DNM_RF-15Year models trained is 0.577, while the mean-test AUC of the best DNM-15Year model discovered through grid searches is 0.862; that is, grid searches brought in a 49.4% performance increase in this case. DFNN was developed based on the ANN, which can be considered a special case of DFNN in that an ANN is just a DFNN that contains only one hidden layer. Both deep learning and grid search became popular long after the ANN was first introduced. The results in Table 3 disclose that a neural network-based method is more sensitive to hyperparameter tunning than some other ML methods such as NB, LR, LASSO, KNN, and SVM; therefore, they help explain why the ANN often performed worse than some other ML methods in earlier years since its invention when grid search was not applied [62,63,64].

We notice from Table 3 that some of the ML methods including LASSO, LR, NB, KNN, and ADB are less affected by grid search. The percentage performance increase in these methods ranges from 0.3% (LASSO) to 10.5% (ADB). LASSO and LR are the two methods least influenced by grid search, with less than 1% performance improvement across all their models. Although these five methods are less sensitive to grid search, some of them are among the top performers. For example, LASSO_RF-15Year ranks number three out of all 15-year models, and LASSO-5Year ranks number five out of all 5-year models; NB-5Year ranks number two and NB_RF-5Year four among all 5-year models, and NB-10Year ranks number four among all 10-year models. In addition, NB-5Year and NB_RF-5Year both rank number one among their peers based on the validation AUCs, as shown in Figure 6, indicating NB tends to do well when handling unseen patients. This is because during the validation process we used the set-aside data that did not participate in model training ang testing. Therefore, when a grid search is not feasible, methods like LASSO and NB should not be bad choices in a similar prediction task.

It is not surprising to see that deep learning performs very well among all the ML methods, with DNM_RF-15Year ranking number one among all 15-year models, and DNM-10Year ranking number three among all 10-year models. It is worth mentioning that some of the other ML methods demonstrate good performance also. For example, the two ensemble methods, XGB and RaF, are both top performers. XGB ranks number one among all other methods when predicting 5-year or 10-year BCM. RaF-5Year ranks number three, RaF-10Year two, and RaF_RF-15Year three, among its peers. These two methods also consistently excel when making predictions for new patients. As shown in Figure 6, which was created using the validation AUCs, both RaF-10Year and RaF_RF-10Year rank number one among their own peers, and XGB_RF-15Year ranks the highest among its peers. Although DFNN, XGB, and RaF all benefit greatly from grid search, XGB and RaF require way less time than DFNN. For example, based on Table 4, it takes about 6216, 13, and 16 min to train DNM_RF-15, XGB_RF-15, and RaF_RF-15, with 6000 HYPESs, respectively. So, when we have very limited time and budget to run grid searches, XGB and RaF are good alternatives for costly deep learning.

The SHAP analyses reveal the relative importance of the predictors. Based on Figure 7, DNM and DNM-RF agree completely that the top three most important features for predicting 15-year BCM are *lymph_node _status*, *age_at_diagnosis*, and *menopausal_status*. From Figure 8, we notice that the least important features such as *HER2*, *Invasive*, and *p53* found in the DNM-15Year model were not even included as predictors in the DNM_RF-15Year model. Recall that the DNM_15Year model was trained purely using the deep learning method, while the predictor inclusion of the DNM_RF-15Year model was solely determined using the BN-based MBIL method, indicating that the least important features found by deep learning were also independently identified by MBIL. Therefore, DFNN and MBIL support the validity of each other in this regard. The top-ranked SHAP features align with known clinical prognostic factors in breast cancer. We found that axillary lymph node involvement, tumor grade, estrogen receptor (ER) status, tumor size, and histological subtype consistently ranked among the most influential predictors across time horizons. These features are well-established in the oncology literature as key determinants of recurrence risk, supporting the clinical face validity of our model. For instance, lymph node positivity is a strong predictor of metastatic spread, while ER positivity is associated with prolonged recurrence windows, often extending beyond 10 years. This alignment between model-derived importance and domain knowledge builds confidence in the model’s decision logic and enhances its clinical interpretability. Furthermore, the use of SHAP enables clinicians to visualize patient-specific contributions, potentially supporting more tailored long-term surveillance or treatment decisions.

Although our grid search process is computationally intensive during model development, once a high-performing model is selected, its application in a clinical setting is computationally light and can be embedded into existing decision support systems. Moreover, reduced model variants or distillation strategies can be employed to streamline deployment.

While our models achieved strong predictive performance using only routinely collected clinical and histopathological data, we acknowledge an important limitation: the exclusion of imaging and molecular biomarker data. These modalities have demonstrated value in stratifying breast cancer subtypes and prognoses in prior studies, and their integration may improve model generalizability, particularly in more heterogeneous patient populations. Our decision to focus solely on clinical data was driven by the goal of developing low-cost, broadly deployable prediction tools that can function in settings lacking advanced molecular diagnostics. Nonetheless, future work should explore the incorporation of radiomic or genomic features to assess potential additive value and to validate model performance across more diverse cohorts.

## 5. Conclusions

This study demonstrates that deep learning, BN-based methods, and grid search are all powerful machine learning tools. Through the coalition of the three, we obtained the best mean-test AUC out of 1,800,000 + ML models that were trained and tested. The DNM_RF model, obtained from this coalition, outperformed all other ML models when predicting the risks of 15-year BCM. The effectiveness of the BN-based MBIL method in identifying risk factors for a disease outcome is further substantiated through this study. The grid search mechanism is shown to be a very powerful prediction optimization method not only for deep learning but also for some of the other ML methods. Surprisingly, some of the ML methods such as the ensemble XGB and *Random Forrests* can outperform deep learning through grid searches, but take “no time” relative to deep learning, while some other ML methods such as BN-based Naïve Bayes, LASSO, LR, and KNN, which are overall less sensitive to grid search, can sometimes excel in prediction also. This suggests that non-deep learning ML methods—despite being tuned via grid search in this study—remain computationally efficient and competitive alternatives to deep learning models for similar prediction tasks, especially when budget is limited or rapid development is needed.

## Figures and Tables

**Table 1:** Case counts and number of predictors of the six LSM datasets.

	Total # of Cases	# of Positive Cases	# of Negative Cases	# of Predictors
LSM-5year	4189	437	3752	31
LSM-10year	1827	572	1255	31
LSM-15year	751	608	143	31
LSM_RF-5year	4189	437	3752	20
LSM_RF-10Year	1827	572	1255	18
LSM_RF-15Year	751	608	143	17

**Table 2:** Side-by-side comparisons of the best all-feature and RF models of the ten ML methods.

5 Year				
Model	Mean-Test AUC	Model	Mean-Test AUC	% Difference
DNM	0.766	DNM_RF	0.750	−2.09%
ADB	0.748	ADB−RF	0.695	−7.09%
NB	0.803	NB_RF	0.773	−3.74%
DT	0.756	DT_RF	0.740	−2.12%
KNN	0.747	KNN_RF	0.725	−2.95%
LASSO	0.772	LASSO_RF	0.719	−6.87%
LR	0.772	LR_RF	0.720	−6.74%
RaF	0.783	RaF_RF	0.755	−3.58%
SVC	0.725	SVC_RF	0.692	−4.55%
XGB	0.804	XGB_RF	0.762	−5.22%
**10 Year**				
**Model**	**Mean-Test AUC**	**Model**	**Mean-Test AUC**	**% Difference**
DNM	0.801	DNM_RF	0.782	−2.37%
ADB	0.763	ADB−RF	0.722	−5.37%
NB	0.801	NB_RF	0.785	−2.00%
DT	0.765	DT_RF	0.747	−2.35%
KNN	0.757	KNN_RF	0.746	−1.45%
LASSO	0.790	LASSO_RF	0.720	−8.86%
LR	0.790	LR_RF	0.722	−8.61%
RaF	0.806	RaF_RF	0.788	−2.23%
SVC	0.792	SVC_RF	0.758	−4.29%
XGB	0.811	XGB_RF	0.791	−2.47%
**15 Year**				
**Model**	**Mean-Test AUC**	**Model**	**Mean-Test AUC**	**Percent Difference**
DNM	0.818	DNM_RF	0.862	5.38%
ADB	0.792	ADB−RF	0.840	6.06%
NB	0.817	NB_RF	0.833	1.96%
DT	0.800	DT_RF	0.786	−1.75%
KNN	0.782	KNN_RF	0.810	3.58%
LASSO	0.804	LASSO_RF	0.847	5.35%
LR	0.805	LR_RF	0.846	5.09%
RaF	0.817	RaF_RF	0.815	−0.24%
SVC	0.807	SVC_RF	0.847	4.96%
XGB	0.814	XGB_RF	0.829	1.84%

**Table 3:** Mean-test AUC of the best model vs. average mean-test AUC of all corresponding models.

			5 Year			
	Average 5 Year	Best 5 Year	% Difference	Average RF 5 Year	Best RF 5 Year	% Difference
DNM	0.542	0.766	41.3%	0.535	0.75	40.2%
ADB	0.611	0.748	22.4%	0.629	0.695	10.5%
NB	0.78	0.803	2.9%	0.756	0.773	2.2%
DT	0.56	0.756	35.0%	0.553	0.74	33.8%
KNN	0.727	0.747	2.8%	0.704	0.725	3.0%
LASSO	0.77	0.772	0.3%	0.717	0.719	0.3%
LR	0.77	0.772	0.3%	0.716	0.72	0.6%
RaF	0.556	0.783	40.8%	0.547	0.755	38.0%
SVC	0.563	0.725	28.8%	0.517	0.692	33.8%
XGBoost	0.594	0.804	35.4%	0.585	0.762	30.3%
			**10 Year**			
	**Average 10 Year**	**Best 10 Year**	**% Difference**	**Average RF 10 Year**	**Best RF 10 Year**	**% Difference**
DNM	0.553	0.801	44.8%	0.553	0.782	41.4%
ADB	0.692	0.763	10.3%	0.691	0.722	4.5%
NB	0.794	0.801	0.9%	0.768	0.785	2.2%
DT	0.556	0.765	37.6%	0.544	0.747	37.3%
KNN	0.744	0.757	1.7%	0.728	0.746	2.5%
LASSO	0.787	0.79	0.4%	0.718	0.72	0.3%
LR	0.787	0.79	0.4%	0.718	0.722	0.6%
RaF	0.549	0.806	46.8%	0.544	0.788	44.9%
SVC	0.605	0.792	30.9%	0.571	0.758	32.7%
XGBoost	0.569	0.811	42.5%	0.559	0.791	41.5%
			**15 Year**			
	**Average 15 Year**	**Best 15 Year**	**% Difference**	**Average RF 15 Year**	**Best RF 15 Year**	**% Difference**
DNM	0.563	0.818	45.3%	0.577	0.862	49.4%
ADB	0.694	0.792	14.1%	0.761	0.84	10.4%
NB	0.785	0.817	4.1%	0.764	0.833	9.0%
DT	0.576	0.8	38.9%	0.59	0.786	33.2%
KNN	0.759	0.782	3.0%	0.767	0.81	5.6%
LASSO	0.799	0.804	0.6%	0.843	0.847	0.5%
LR	0.799	0.805	0.8%	0.843	0.846	0.4%
RaF	0.56	0.817	45.9%	0.544	0.815	49.8%
SVC	0.639	0.807	26.3%	0.676	0.847	25.3%
XGBoost	0.523	0.814	55.6%	0.518	0.829	60.0%

**Table 4:** Comparison of grid search running time (in minutes) between corresponding all-feature and RF models. Each grid search trained 6000 models.

Model	5 Year	RF 5 Year	10 Year	RF 10 Year	15 Year	RF 15 Year
DNM	85,932.83	85,402.63	31,053.83	31,110.23	6308.75	6215.888
ADB	40.85	1798.62	15.55	1971.38	10.33	563.555
NB	0.6	0.45	0.47	0.3	0.32	0.23
DT	0.33	0.28	0.22	0.17	0.18	0.13
KNN	22.77	20.17	5.15	4.37	1.4	1.28
LASSO	1.12	0.7	0.78	0.33	0.57	0.25
LR	0.92	0.58	0.8	0.38	0.63	0.28
RaF	25.65	25.32	20.73	15.3	17.53	16.35
SVC	9.7	8.58	4.02	3.82	0.9	0.68
XGB	26.08	19.3	14.58	10.55	14.40	12.58

## Data Availability

The data used in this study are available at datadryad.org (DOI: 10.5061/dryad.64964m0).

## Supplementary Materials

The following supporting information can be downloaded at https://www.mdpi.com/article/10.3390/cancers17152515/s1, Table S1: The variables of the LSM datasets; Table S2: Predictors in the LSM_RF-5Year Dataset; Table S3: Predictors in the LSM_RF-10Year Dataset; Table S4: Predictors in the LSM_RF-15 Year Dataset; Table S5: The ML hyperparameters and their values given to the RGSP; Figure S1: SHAP summary plots for the two best DFNN-based models concerning 5-year BCM; Figure S2: SHAP summary plots for the two best *Naïve Bayes* models concerning 5-year BCM; Figure S3: SHAP summary plots for the two best *Random Forests* models concerning 5-year BCM; Figure S4: SHAP summary plots for the best two XGBoost Models concerning 5-year BCM; Figure S5: SHAP summary plots for the two best DFNN-based models concerning 10-year BCM; Figure S6: SHAP summary plots for the two best *Naïve Bayes* models concerning 10-year BCM; Figure S7: SHAP summary plots for the two best *Random Forests* models concerning10-year BCM; Figure S8: SHAP summary plots for the two best XGBoost models concerning 10-year BCM; Figure S9: SHAP summary plots for the two best LASSO models concerning 15-year BCM; Figure S10: SHAP summary plots for the two best *Logistic Regression* models concerning 15-year BCM; Figure S11: SHAP summary plots for the two best SVC models concerning 15-year datasets; Figure S12: Procedure chart for identifying a best model through grid search; Figure S13: The calibration curve and Brier Score for the best 5_year model (XGB_5Year); Figure S14: The calibration curve and Brier Score for the best 10_year model (XGB_10Year); Figure S15: The calibration curve and Brier Score for the best 15_year model (DNM_RF_15Year).

## Abbreviations

BCM	breast cancer metastasis
MBIL	Markov blanket and interactive risk factor learner
BN	Bayesian network
DAG	directed acyclic graph
DFNN	Deep feed forward neural network
DNNs	Deep neural networks
HYPES	hyperparameter setting
P-HYPESs	pool of hyperparameter settings
RGS	Randomized Grid Search
RGS_HSG	Hyperparameter Setting Generator
CV	cross validation
RGSP	Randomized Grade Search Package
DNM	deep feed-forward neural network models for breast cancer metastasis
RF	risk factor
ML	machine learning
BDeu	Bayesian Dirichlet equivalent uniform
EHR	electronic health records
TNBC	triple negative breast cancer
SHGS	Single Hyperparameter Grid Searches
ROC curve	receiver operator characteristic curve
TPR	true positive rate
FPR	false positive rate
