# Supplementary material for: Leveraging Deep Learning, Grid Search, and Bayesian Networks to Predict Distant Recurrence of Breast Cancer
Source: Cancers (Basel). 2025 Jul 30;17(15):2515. doi: 10.3390/cancers17152515 (PMC12346417; doi:10.3390/cancers17152515)
Supplement: Supplementary file 1 [file cancers-17-02515-s001.zip › cancers-3729145-supplementary.pdf]

Supplement

Table S1. The variables of the LSM datasets.

Abbreviation	Variable Name	Description	Values
RAC	*race*	race of patient	white, black, Asian, American Indian or Alaskan native, native Hawaiian or other Pacific islander
ETH	*ethnicity*	ethnicity of patient	not Hispanic, Hispanic
SMO	*smoking*	smoking history of patient	ex smoker, non smoker, cigarettes, chewing tobacco, cigar
ALC	*alcohol usage*	alcohol usage of patient	moderate, no use, use but nos (non otherwise specified), former user, heavy user
FAM	*family history*	family history of cancer	cancer, no cancer, breast cancer, other cancer, cancer but nos
AGE	*age_at_diagnosis*	age at diagnosis of the disease	0-49, 50-69, >69
MEN	*menopausal_status*	inferred menopausal status	pre, post
SID	*side*	side of tumor	left, right
TNE	*TNEG*	triple negative status in terms of patient being ER, PR, and HER2 negative	yes, no
ER	*ER*	estrogen receptor expression	neg, pos, low pos
ERP	*ER_percent*	percent of cell stain pos for ER receptors	0-20, 20-90, 90-100
PR	*PR*	progesterone receptor expression	neg, pos, low pos
PRP	*PR_percent*	percent of cell stain pos for PR receptors	0-20, 20-90, 90-100
P53	*P53*	whether P53 is mutated	neg, pos, low pos
HER	*HER2*	HER2 expression	neg, pos
TTN	*t_tnm_stage*	prime tumor stage in TNM system	0, 1, 2, 3, 4, IS, 1mic, X
NTN	*n_tnm_stage*	# of nearby cancerous lymph nodes	0, 1, 2, 3, 4, X
STA	*stage*	composite of size and # positive nodes	0, 1, 2, 3
LYR	*lymph_nodes_removed*	number of lymph nodes removed	0-11, 12-22, > 22
LYP	*lymph_nodes_positive*	number of positive lymph nodes	0, 1-8, >8
LYS	*lymph_node_status*	patient had any positive lymph nodes	neg, pos
HI1	*histology*	tumor histology	lobular, duct
SIZ	*size*	size of tumor in mm	0-32, 32-70, >70
GRA	*grade*	grade of disease	1, 2, 3
INV	*invasive*	whether tumor is invasive	yes, no
HI2	*histology2*	tumor histology subtypes	IDC, DCIS, ILC, NC
INL	*invasive_tumor_location*	where invasive tumor is located	mixed duct and lobular, duct, lobular, none
DCI	*DCIS_level*	type of ductal carcinoma in situ	solid, apocrine, cribriform, dcis, comedo, papillary, micropapillary
REE	*re_excision*	removal of an additional margin of tissue	yes, no
SUR	*surgical_margins*	whether residual tumor	res. tumor, no res. tumor,

			no primary site surgery
MRI	*MRIs_60_surgery*	MRIs within 60 days of surgery	yes, no
	Metastasis	This is the outcome variable	yes, no

Table S2. Predictors in the LSM_RF-5Year Dataset.

	Predictors	Description	Values
1	*race*	*race of patient*	*white, black, Asian, American Indian or Alaskan native, native Hawaiian or other Pacific islander*
2	*smoking*	smoking history of patient	ex smoker, non smoker, cigarettes, chewing tobacco, cigar
3	*family history*	family history of cancer	cancer, no cancer, breast cancer, other cancer, cancer but nos
4	*age_at_diagnosis*	age at diagnosis of the disease	0-49, 50-69, >69
5	*TNEG*	triple negative status in terms of patient being ER, PR, and HER2 negative	yes, no
6	*ER*	estrogen receptor expression	neg, pos, low pos
7	*ER_percent*	percent of cell stain pos for ER receptors	0-20, 20-90, 90-100
8	*PR*	progesterone receptor expression	neg, pos, low pos
9	*PR_percent*	percent of cell stain pos for PR receptors	0-20, 20-90, 90-100
10	*P53*	*P53*	*whether P53 is mutated*
11	*HER2*	HER2 expression	neg, pos
12	*t_tnm_stage*	*prime tumor stage in TNM system*	*0, 1, 2, 3, 4, IS, 1mic, X*
13	*n_tnm_stage*	*# of nearby cancerous lymph nodes*	*0, 1, 2, 3, 4, X*
14	*stage*	*composite of size and # positive nodes*	*0, 1, 2, 3*
15	*lymph_nodes_positive*	number of positive lymph nodes	0, 1-8, >8
16	*histology*	tumor histology	lobular, duct
17	*size*	*size of tumor in mm*	*0-32, 32-70, >70*
18	*invasive_tumor_location*	*where invasive tumor is located*	*mixed duct and lobular, duct, lobular, none*
19	*DCIS_level*	*type of ductal carcinoma in situ*	*solid, apocrine, cribriform, dcis, comedo, papillary, micropapillary*
20	*surgical_margins*	whether residual tumor	res. tumor, no res. tumor,<br>no primary site surgery

Table S3. Predictors in the LSM_RF-10Year Dataset.

	Predictors	Description	Values
1	*ethnicity*	ethnicity of patient	not Hispanic, Hispanic
2	*smoking*	smoking history of patient	ex smoker, non smoker, cigarettes, chewing tobacco, cigar
3	*alcohol usage*	alcohol usage of patient	moderate, no use, use but nos (non otherwise specified), former user, heavy user
4	*family history*	family history of cancer	cancer, no cancer, breast cancer, other cancer, cancer but nos
5	*age_at_diagnosis*	age at diagnosis of the disease	0-49, 50-69, >69
6	*TNEG*	triple negative status in terms of	yes, no

		patient being ER, PR, and HER2 negative	
7	*ER*	estrogen receptor expression	neg, pos, low pos
8	*ER_percent*	percent of cell stain pos for ER receptors	0-20, 20-90, 90-100
9	*PR*	progesterone receptor expression	neg, pos, low pos
10	*PR_percent*	percent of cell stain pos for PR receptors	0-20, 20-90, 90-100
11	*HER2*	HER2 expression	neg, pos
12	*n_tnm_stage*	# of nearby cancerous lymph nodes	0, 1, 2, 3, 4, X
13	*stage*	composite of size and # positive nodes	0, 1, 2, 3
14	*lymph_nodes_positive*	number of positive lymph nodes	0, 1-8, >8
15	*histology*	tumor histology	lobular, duct
16	*grade*	grade of disease	1, 2, 3
17	*DCIS_level*	type of ductal carcinoma in situ	solid, apocrine, cribriform, dcis, comedo, papillary, micropapillary
18	*surgical_margins*	whether residual tumor	res. tumor, no res. tumor,<br>no primary site surgery

Table S4. Predictors in the LSM_RF-15 Year Dataset.

	Predictors	Description	Values
1	*race*	race of patient	white, black, Asian, American Indian or Alaskan native, native Hawaiian or other Pacific islander
2	*alcohol usage*	alcohol usage of patient	moderate, no use, use but nos (non otherwise specified), former user, heavy user
3	*age_at_diagnosis*	age at diagnosis of the disease	0-49, 50-69, >69
4	*menopausal_status*	inferred menopausal status	pre, post
5	*ER*	estrogen receptor expression	neg, pos, low pos
6	*ER_percent*	percent of cell stain pos for ER receptors	0-20, 20-90, 90-100
7	*t_tnm_stage*	prime tumor stage in TNM system	0, 1, 2, 3, 4, IS, 1mic, X
8	*n_tnm_stage*	# of nearby cancerous lymph nodes	0, 1, 2, 3, 4, X
9	*stage*	composite of size and # positive nodes	0, 1, 2, 3
10	*lymph_node_status*	patient had any positive lymph nodes	neg, pos
11	*size*	size of tumor in mm	0-32, 32-70, >70
12	*grade*	grade of disease	1, 2, 3
13	*histology2*	tumor histology subtypes	IDC, DCIS, ILC, NC
14	*invasive_tumor_location*	where invasive tumor is located	mixed duct and lobular, duct, lobular, none
15	*re_excision*	removal of an additional margin of tissue	yes, no
16	*surgical_margins*	whether residual tumor	res. tumor, no res. tumor,<br>no primary site surgery
17	*histology*	tumor histology	lobular, duct

Table S5. The ML hyperparameters and their values given to the RGSP.

Method	Hyperparameter	Description	Values
DFNN	Epochs	Number of times model is trained by the full training dataset	5 ~ 1001, step size 3
	Batch size	Number of samples that are processed together in a single forward and backward pass during training	1 to the # of datapoints in a dataset
	Learning rate	Control the learning and parameter update speed during optimization	0.001 ~ 0.3, Step size 0.001
	Dropout rate	Mitigate overfitting and training time by randomly ignoring nodes	0 ~ 0.9, Step size 0.01
	Momentum	Speed up optimization by incorporating historical gradients into parameter updates. Momentum is exclusively applicable within the SGD optimizer.	0.1 ~ 0.9, Step size 0.01
	decay	Iterative decay of the learning rate by applying a decreasing factor at each epoch	0 ~ 0.3, Step size 0.001
	L1 weight	Control the strength of L1 regularization in model training	0 ~ 0.3, Step size 0.001
	L2 weight	Control the strength of L2 regularization in model training	0 ~ 0.3, Step size 0.001
	# of Hidden Layers	The depth of a DNN model	1,2,3,4
	# of Hidden Nodes	Number of neurons in a hidden layer	All integers from 1 to the number of datapoints of each dataset
	Optimizer	Optimizes model parameters during training process towards minimizing the loss	SGD, Adam, Adagrad, Nadam, Adamax
	Weight Initializer	A technique employed to assign initial values to the weights of the connections between neurons in a neural network	Constant, Glorot_normal, Glorot_uniform, He_normal, He_uniform
	Input Layer Activation Function	A function applied to the input data of a neural network's input layer	Relu, Sigmoid, Softmax, Tanh
	Hidden Layer Activation Function	A function applied to the output of a hidden layer in a neural network	Relu, Sigmoid, Softmax, Tanh
	Output Layer Activation Function	A function applied to the output of a neural network's output layer, producing the final prediction of the network.	Sigmoid
	Loss Function	A method of evaluating the dissimilarity between the predicted output of a model and the actual values	Binary_crossentropy
AdaBoost	Base_estimator	The model that AdaBoost will enhance through iterative updates	Logistic Regression, Random Forest, Decision Tree
	n_estimators	The maximum number of estimators at which boosting is terminated	1 ~ 1001, step size 1
	Learning rate	The weight applied to each classifier at each boosting iteration	0.001 ~ 0.101, step size 0.001
	algorithm	The technique AdaBoost uses to update weights and make predictions	SAMME

Naïve Bayes	alpha	The additive smoothing parameter	0.00001 ~ 100, step size 0.00001
	Min_categories	The minimum number of categories per feature	5 ~ 16, step size 1
	Fit_prior	Defines whether to learn class prior probabilities or not	True, false
Decision tree	Criterion	The function to measure the quality of a split	Gini, Entropy, Log_loss
	Splitter	The strategy used to choose the split at each node	Best, Random
	Max_depth	The maximum depth of the tree	1 ~ 100, step size 1
	Min_samples_split	The minimum number of samples required to split an internal node	2 to the # of datapoints in a dataset, step size 1
	Min_samples_leaf	The minimum number of samples required to be at a leaf node	2 to half of the # of datapoints in a dataset, step size 1
	Min_weight_fraction_leaf	The minimum weighted fraction of the sum total of weights	0 ~ 0.5, step size 0.001
	Max_features	The number of features to consider when looking for the best split	1 ~ 17, step size 1
	Max_leaf_nodes	The maximum number of leaf nodes a tree can have	2 to the # of datapoints in a dataset, step size 1
	Min_impurity_decrease	The hyperparameter controls the minimum reduction in impurity required for a split to be made at a node	0 ~ 0.01, step size 0.001
	Class_weight	The weights associated with classes in the form	Balanced, None
KNN	N_neighbors	Number of neighbors to use	1 ~ 100, step size 1
	Weights	The weight function used in prediction	Uniform, Distance, None
	Algorithm	The algorithm used to compute the nearest neighbors	Auto, Ball_tree, Kd_tree, Brute
	Leaf_size	The parameter that affects the speed of the construction and query	1 to the # of datapoints in a dataset, step size 1
LASSO	Max_iter	The maximum number of iterations	5 ~ 1000, step size 1
	Tol	The tolerance for the optimization	0.00001 ~ 0.01, step size 0.00005
	C	The inverse of regularization strength	0 ~ 100,step size 0.00005
	Class_weight	The parameter that adjusts the weights	Balanced, None
	Solver	The algorithm used by the optimization problem	Liblinear, Saga
Logistic Regression	Max_iter	The maximum number of iterations taken for the solvers to converge	5 ~ 1000, step size 1
	Tol	The tolerance for stopping criteria	0.00001 ~ 0.01, step size 0.00005
	C	The inverse of regularization strength	0 ~ 100, step size 0.005
	Class_weight	The weights associated with classes in the form	Balanced, none
	Solver	The algorithm that used in the optimization probem	None, L1, L2, Elasticnet
Random Forest	N_estimators	The number of tress in the forest	1 ~ 1000, step size 1
	Criterion	The function to measure the quality of a split	Gini, Entropy, Log_loss
	Max_depth	The maximum depth of the tree	1 ~ 101,step size 1

	Min_samples_split	The minimum number of samples required to split an internal node	2 to the # of datapoints in a dataset, step size 1
	Min_samples_leaf	The minimum number of samples	1 to half of the # of datapoints in a dataset, step size 1
	Min_weight_fraction_leaf	The minimum weighted fraction of the sum total of weights	0 ~ 0.5,step size 0.001
	Max_features	The number of features to consider when looking for the best split	1 ~ 17,step size 1
	Max_leaf_nodes	The maximum number of leaf nodes a tree can have	2 to half of the # of datapoints in a dataset, step size 1
	Min_impurity_decrease	The hyperparameter controls the minimum reduction in impurity required for a split to be made at a node	0 ~ 0.01,step size 0.001
	Class_weight	The weights associated with classes in the form	Balanced, None
SVC	C	The regularization parameter	0 ~ 100,step size 0.005
	Kernel	The hyperparameter that specifies the kernel type to be used in the algorithm	Linear, Poly, Rbf, Sigmoid
	Degree	The degree of the polynomial kernel function	0 ~ 5,step size 1
	Gamma	The kernel coefficient	Scale, Auto
	Shrinking	Defines whether to use shrinking heuristic	True, false
	Tol	The tolerance for stopping criterion	0.00001 ~ 0.01,step size 0.00005
	Class_weight	The weights associated with classes in the form	Balanced, None
	Max_iter	The maximum number of iterations taken for the solvers to converge	5 ~ 1000, step size 1
XGBoost	Booster	The hyperparameter defines which booster to use	Gbtree, Gblinear, Dart
	Eta	The hyperparameter defines step size shrinkage for tree booster	0.001 ~ 0.1,step size 0.001
	Gamma	The minimum loss reduction required to make a further partition on a leaf node of the tree for tree booster	0 ~ 10, step size 0.01
	Max_depth	The maximum depth of a tree for tree booster	1 ~ 100, step size 1
	Subsample	The hyperparameter defines the subsample ratio of the training instances	0.01~ 1, step size 0.01
	Sampling_method	The method to use to sample the training instances	Uniform, Gradient_based
	Alpha	The L1 regularization term on weights	0 ~ 100,s tep size 0.00001
	Lambda	The L2 regularization term on weights	0 ~ 100, step size 0.00001
	Tree_method	The tree construction algorithm used in XGBoost	Auto, Exact, Approx, Hist
	Objective	The hyperparameter specifies the learning objective	Binary:logistic

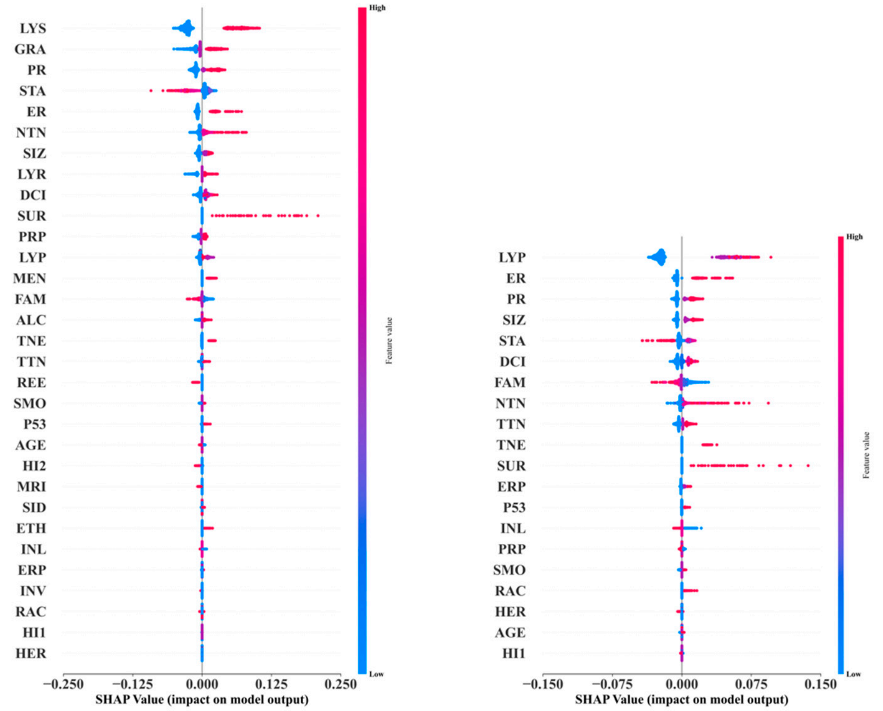

(a) **(b)**

Figure S1. SHAP summary plots for the two best DFNN-based models concerning 5-year BCM: (a) DNM-5Year; and (b) DNM_RF-5Year

AGE*: age at diagnosis of the disease;* ***ALC****: alcohol usage;* ***DCI****: type of ductal carcinoma in situ;* ***ER****: estrogen receptor expression;* ***ERP****: percent of cell stain pos for ER receptors;* ***ETH****: ethnicity;* ***FAM****: family history of cancer;* ***GRA****: grade of disease;* ***HER****: HER2 expression;* ***HI1****: tumor histology****;*** ***HI2****: tumor histology subtypes;* ***INL****: where invasive tumor is located;* ***INV****: whether tumor is invasive;* ***LYP****: number of positive lymph nodes;* ***LYR****: number of lymph nodes removed;* ***LYS****: patient had any positive lymph nodes;* ***MEN****: inferred menopausal status;* ***MRI****: MRIs within 60 days of surgery****;*** ***NTN****: number of nearby cancerous lymph nodes;* ***PR****: progesterone receptor expression;* ***PRP****: percent of cell stain pos for PR receptors;* ***P53****: whether P53 is mutated;* ***RAC****: race;* ***REE****: removal of an additional margin of tissue;* ***SID****: side of tumor;* ***SIZ****: size of tumor in mm;* ***SMO****: smoking;* ***STA****: composite of size and # positive nodes;* ***SUR****: whether residual tumor;* ***TNE****: triple negative status in terms of patient being ER, PR, and HER2 negative;* ***TTN****: prime tumor stage in TNM system.*

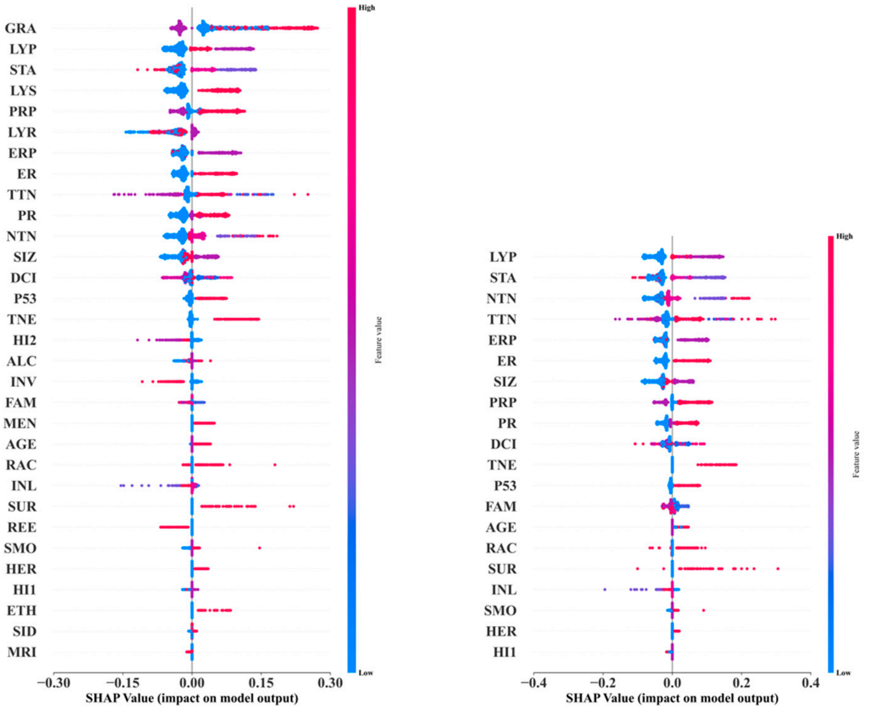

(a) **(b)**

Figure S2. SHAP summary plots for the two best *Naïve Bayes* models concerning 5-year BCM: (a)NB-5Year; and (b) NB_RF-5Year

AGE: *age at diagnosis of the disease;* ***ALC***: *alcohol usage;* ***DCI***: *type of ductal carcinoma in situ;* ***ER***: *estrogen receptor expression;* ***ERP***: *percent of cell stain pos for ER receptors;* ***ETH***: *ethnicity;* ***FAM***: *family history of cancer;* ***GRA***: *grade of disease;* ***HER***: *HER2 expression;* ***HI1***: *tumor histology**;** **HI2***: *tumor histology subtypes;* ***INL***: *where invasive tumor is located;* ***INV***: *whether tumor is invasive;* ***LYP***: *number of positive lymph nodes;* ***LYR***: *number of lymph nodes removed;* ***LYS***: *patient had any positive lymph nodes;* ***MEN***: *inferred menopausal status;* ***MRI***: *MRIs within 60 days of surgery**;** **NTN***: *number of nearby cancerous lymph nodes;* ***PR***: *progesterone receptor expression;* ***PRP***: *percent of cell stain pos for PR receptors;* ***P53***: *whether P53 is mutated;* ***RAC***: *race;* ***REE***: *removal of an additional margin of tissue;* ***SID***: *side of tumor;* ***SIZ***: *size of tumor in mm;* ***SMO***: *smoking;* ***STA***: *composite of size and # positive nodes;* ***SUR***: *whether residual tumor;* ***TNE***: *triple negative status in terms of patient being ER, PR, and HER2 negative;* ***TTN***: *prime tumor stage in TNM system.*

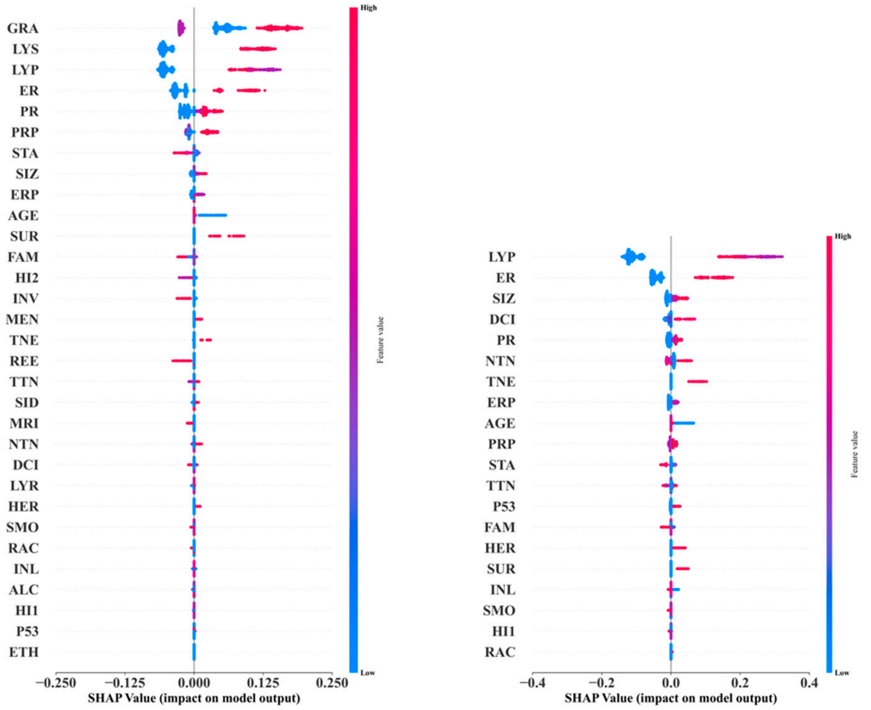

(a) **(b)**

Figure S3. SHAP summary plots for the two best *Random Forests* models concerning 5-year BCM: (a) RaF-5Year; and (b) RaF_RF-5Year

AGE*: age at diagnosis of the disease;* ***ALC****: alcohol usage;* ***DCI****: type of ductal carcinoma in situ;* ***ER****: estrogen receptor expression;* ***ERP****: percent of cell stain pos for ER receptors;* ***ETH****: ethnicity;* ***FAM****: family history of cancer;* ***GRA****: grade of disease;* ***HER****: HER2 expression;* ***HI1****: tumor histology****;*** ***HI2****: tumor histology subtypes;* ***INL****: where invasive tumor is located;* ***INV****: whether tumor is invasive;* ***LYP****: number of positive lymph nodes;* ***LYR****: number of lymph nodes removed;* ***LYS****: patient had any positive lymph nodes;* ***MEN****: inferred menopausal status;* ***MRI****: MRIs within 60 days of surgery****;*** ***NTN****: number of nearby cancerous lymph nodes;* ***PR****: progesterone receptor expression;* ***PRP****: percent of cell stain pos for PR receptors;* ***P53****: whether P53 is mutated;* ***RAC****: race;* ***REE****: removal of an additional margin of tissue;* ***SID****: side of tumor;* ***SIZ****: size of tumor in mm;* ***SMO****: smoking;* ***STA****: composite of size and # positive nodes;* ***SUR****: whether residual tumor;* ***TNE****: triple negative status in terms of patient being ER, PR, and HER2 negative;* ***TTN****: prime tumor stage in TNM system.*

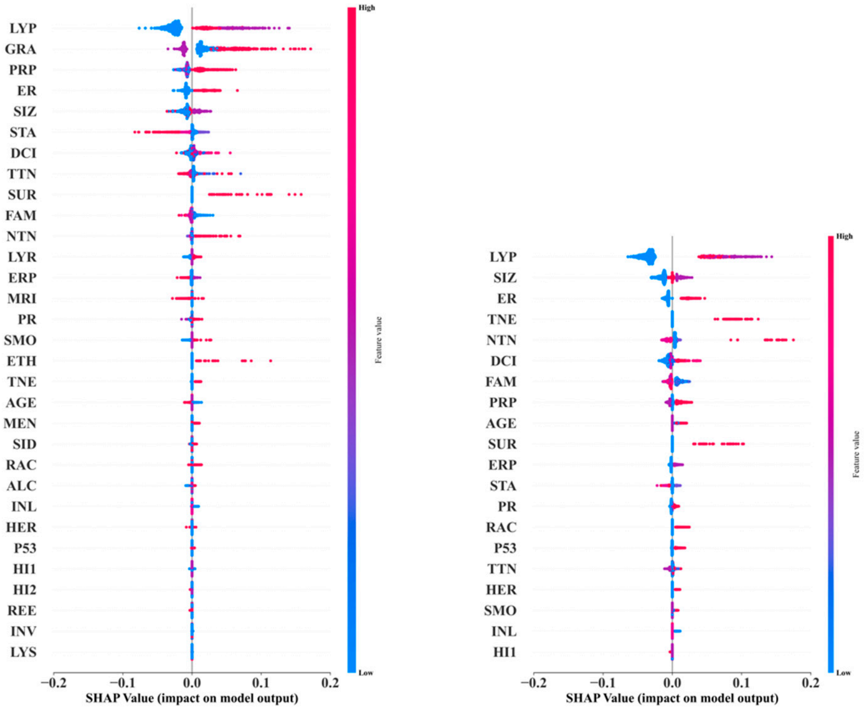

(a) **(b)**

Figure S4. SHAP summary plots for the best two XGBoost Models concerning 5-year BCM
(a) XGB-5Year; and (b) XGB_RF-5Year

AGE*: age at diagnosis of the disease;* ***ALC****: alcohol usage;* ***DCI****: type of ductal carcinoma in situ;* ***ER****: estrogen receptor expression;* ***ERP****: percent of cell stain pos for ER receptors;* ***ETH****: ethnicity;* ***FAM****: family history of cancer;* ***GRA****: grade of disease;* ***HER****: HER2 expression;* ***HI1****: tumor histology****;*** ***HI2****: tumor histology subtypes;* ***INL****: where invasive tumor is located;* ***INV****: whether tumor is invasive;* ***LYP****: number of positive lymph nodes;* ***LYR****: number of lymph nodes removed;* ***LYS****: patient had any positive lymph nodes;* ***MEN****: inferred menopausal status;* ***MRI****: MRIs within 60 days of surgery****;*** ***NTN****: number of nearby cancerous lymph nodes;* ***PR****: progesterone receptor expression;* ***PRP****: percent of cell stain pos for PR receptors;* ***P53****: whether P53 is mutated;* ***RAC****: race;* ***REE****: removal of an additional margin of tissue;* ***SID****: side of tumor;* ***SIZ****: size of tumor in mm;* ***SMO****: smoking;* ***STA****: composite of size and # positive nodes;* ***SUR****: whether residual tumor;* ***TNE****: triple negative status in terms of patient being ER, PR, and HER2 negative;* ***TTN****: prime tumor stage in TNM system.*

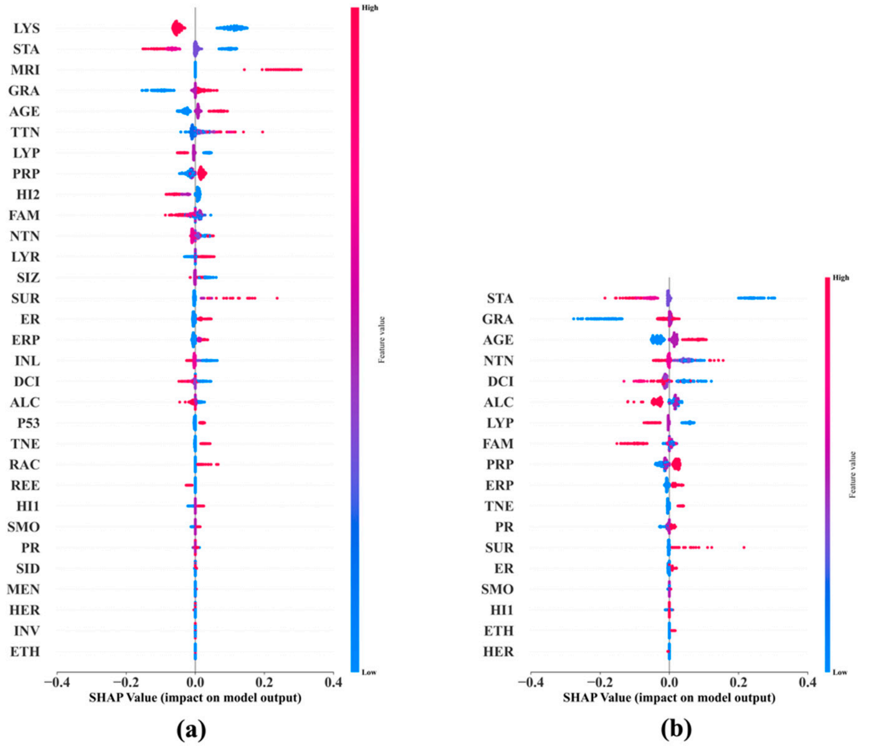

Figure S5. SHAP summary plots for the two best DFNN-based models concerning 10-year BCM
(a) DNM-10Year; and (b) DNM_RF-10Year

AGE*: age at diagnosis of the disease;* ***ALC****: alcohol usage;* ***DCI****: type of ductal carcinoma in situ;* ***ER****: estrogen receptor expression;* ***ERP****: percent of cell stain pos for ER receptors;* ***ETH****: ethnicity;* ***FAM****: family history of cancer;* ***GRA****: grade of disease;* ***HER****: HER2 expression;* ***HI1****: tumor histology;* ***HI2****: tumor histology subtypes;* ***INL****: where invasive tumor is located;* ***INV****: whether tumor is invasive;* ***LYP****: number of positive lymph nodes;* ***LYR****: number of lymph nodes removed;* ***LYS****: patient had any positive lymph nodes;* ***MEN****: inferred menopausal status;* ***MRI****: MRIs within 60 days of surgery;* ***NTN****: number of nearby cancerous lymph nodes;* ***PR****: progesterone receptor expression;* ***PRP****: percent of cell stain pos for PR receptors;* ***P53****: whether P53 is mutated;* ***RAC****: race;* ***REE****: removal of an additional margin of tissue;* ***SID****: side of tumor;* ***SIZ****: size of tumor in mm;* ***SMO****: smoking;* ***STA****: composite of size and # positive nodes;* ***SUR****: whether residual tumor;* ***TNE****: triple negative status in terms of patient being ER, PR, and HER2 negative;* ***TTN****: prime tumor stage in TNM system.*

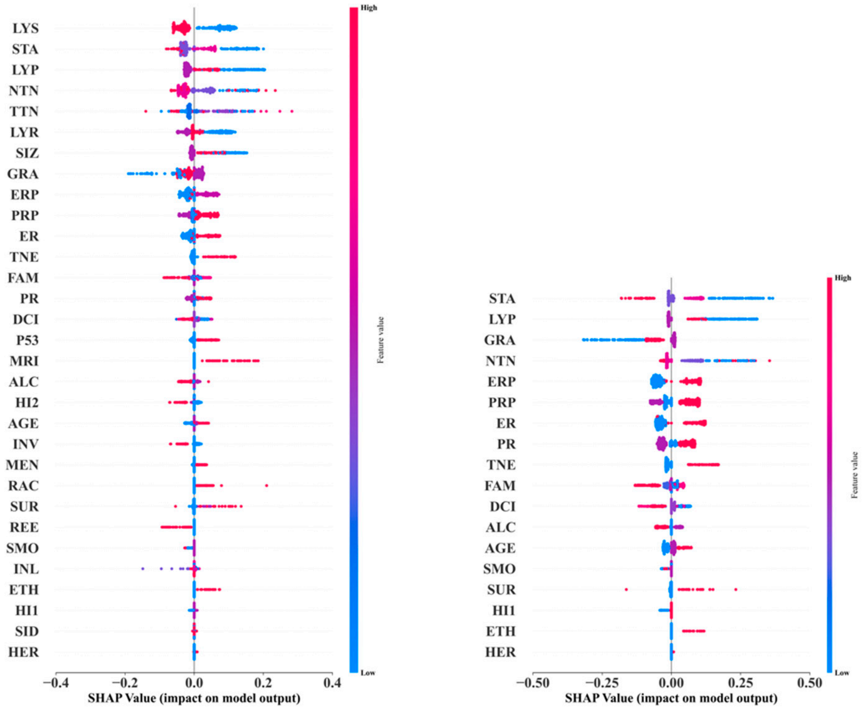

(a) **(b)**

Figure S6. SHAP summary plots for the two best *Naïve Bayes* models concerning 10-year BCM
(a) NB-10Year; and (b) NB_RF-10Year

AGE*: age at diagnosis of the disease;* ***ALC****: alcohol usage;* ***DCI****: type of ductal carcinoma in situ;* ***ER****: estrogen receptor expression;* ***ERP****: percent of cell stain pos for ER receptors;* ***ETH****: ethnicity;* ***FAM****: family history of cancer;* ***GRA****: grade of disease;* ***HER****: HER2 expression;* ***HI1****: tumor histology;* ***HI2****: tumor histology subtypes;* ***INL****: where invasive tumor is located;* ***INV****: whether tumor is invasive;* ***LYP****: number of positive lymph nodes;* ***LYR****: number of lymph nodes removed;* ***LYS****: patient had any positive lymph nodes;* ***MEN****: inferred menopausal status;* ***MRI****: MRIs within 60 days of surgery;* ***NTN****: number of nearby cancerous lymph nodes;* ***PR****: progesterone receptor expression;* ***PRP****: percent of cell stain pos for PR receptors;* ***P53****: whether P53 is mutated;* ***RAC****: race;* ***REE****: removal of an additional margin of tissue;* ***SID****: side of tumor;* ***SIZ****: size of tumor in mm;* ***SMO****: smoking;* ***STA****: composite of size and # positive nodes;* ***SUR****: whether residual tumor;* ***TNE****: triple negative status in terms of patient being ER, PR, and HER2 negative;* ***TTN****: prime tumor stage in TNM system.*

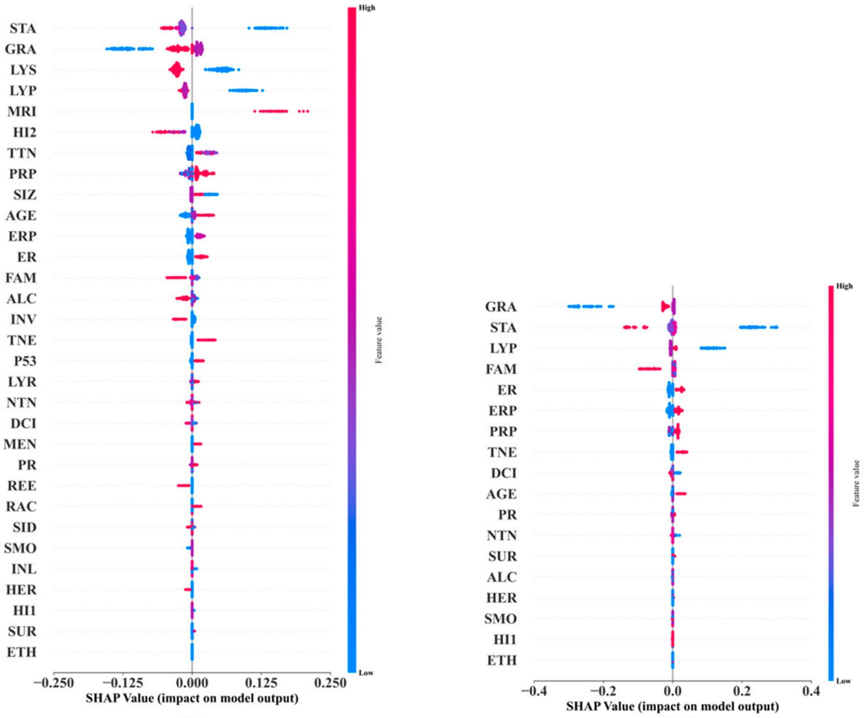

(a) **(b)**

Figure S7. SHAP summary plots for the two best *Random Forests* models concerning10-year BCM (a) RaF-10Year; and (b) RaF_RF-10Year

AGE: *age at diagnosis of the disease;* ***ALC***: *alcohol usage;* ***DCI***: *type of ductal carcinoma in situ;* ***ER***: *estrogen receptor expression;* ***ERP***: *percent of cell stain pos for ER receptors;* ***ETH***: *ethnicity;* ***FAM***: *family history of cancer;* ***GRA***: *grade of disease;* ***HER***: *HER2 expression;* ***HI1***: *tumor histology;* ***HI2***: *tumor histology subtypes;* ***INL***: *where invasive tumor is located;* ***INV***: *whether tumor is invasive;* ***LYP***: *number of positive lymph nodes;* ***LYR***: *number of lymph nodes removed;* ***LYS***: *patient had any positive lymph nodes;* ***MEN***: *inferred menopausal status;* ***MRI***: *MRIs within 60 days of surgery;* ***NTN***: *number of nearby cancerous lymph nodes;* ***PR***: *progesterone receptor expression;* ***PRP***: *percent of cell stain pos for PR receptors;* ***P53***: *whether P53 is mutated;* ***RAC***: *race;* ***REE***: *removal of an additional margin of tissue;* ***SID***: *side of tumor;* ***SIZ***: *size of tumor in mm;* ***SMO***: *smoking;* ***STA***: *composite of size and # positive nodes;* ***SUR***: *whether residual tumor;* ***TNE***: *triple negative status in terms of patient being ER, PR, and HER2 negative;* ***TTN***: *prime tumor stage in TNM system.*

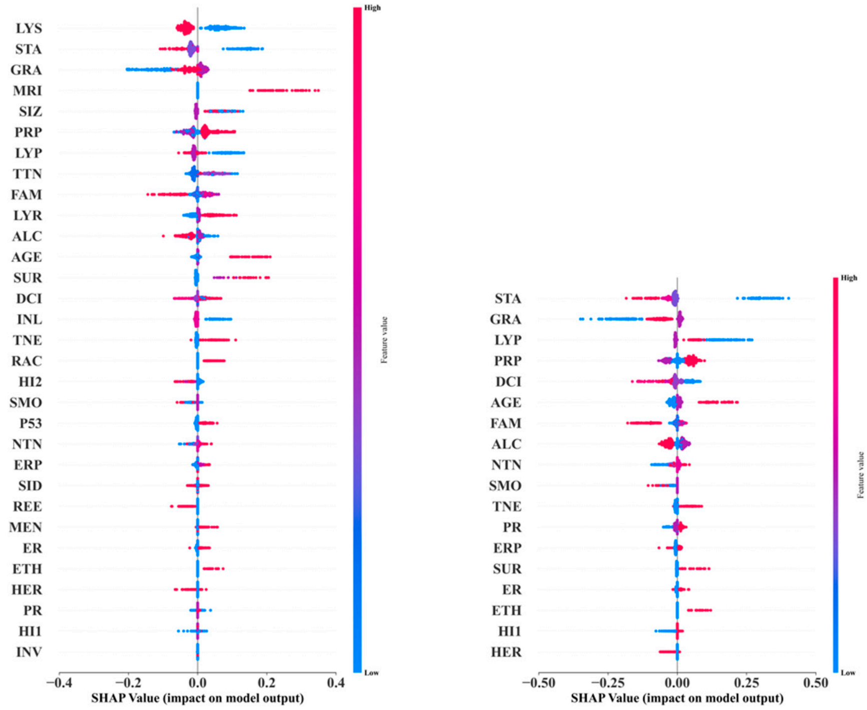

(a) **(b)**

Figure S8. SHAP summary plots for the two best XGBoost models concerning 10-year BCM
(a) XGB-10Year; and (b) XGB_RF-10Year

AGE*: age at diagnosis of the disease;* ***ALC****: alcohol usage;* ***DCI****: type of ductal carcinoma in situ;* ***ER****: estrogen receptor expression;* ***ERP****: percent of cell stain pos for ER receptors;* ***ETH****: ethnicity;* ***FAM****: family history of cancer;* ***GRA****: grade of disease;* ***HER****: HER2 expression;* ***HI1****: tumor histology;* ***HI2****: tumor histology subtypes;* ***INL****: where invasive tumor is located;* ***INV****: whether tumor is invasive;* ***LYP****: number of positive lymph nodes;* ***LYR****: number of lymph nodes removed;* ***LYS****: patient had any positive lymph nodes;* ***MEN****: inferred menopausal status;* ***MRI****: MRIs within 60 days of surgery;* ***NTN****: number of nearby cancerous lymph nodes;* ***PR****: progesterone receptor expression;* ***PRP****: percent of cell stain pos for PR receptors;* ***P53****: whether P53 is mutated;* ***RAC****: race;* ***REE****: removal of an additional margin of tissue;* ***SID****: side of tumor;* ***SIZ****: size of tumor in mm;* ***SMO****: smoking;* ***STA****: composite of size and # positive nodes;* ***SUR****: whether residual tumor;* ***TNE****: triple negative status in terms of patient being ER, PR, and HER2 negative;* ***TTN****: prime tumor stage in TNM system.*

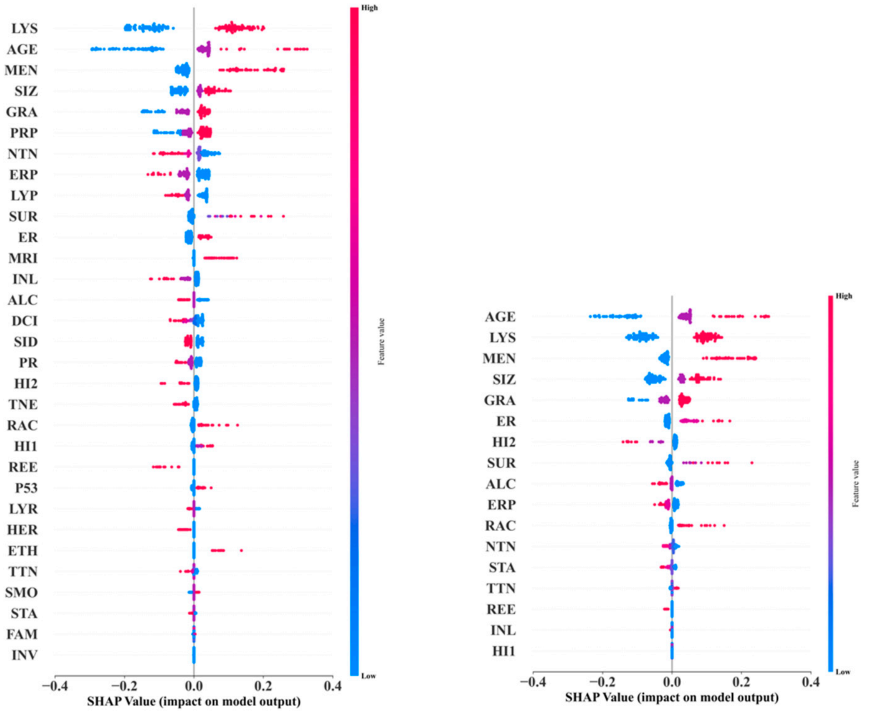

(a) **(b)**

Figure S9. SHAP summary plots for the two best LASSO models concerning 15-year BCM
(a) LASSO-15Year; and (b) LASSO_RF-15Year

AGE: *age at diagnosis of the disease;* ***ALC***: *alcohol usage;* ***DCI***: *type of ductal carcinoma in situ;* ***ER***: *estrogen receptor expression;* ***ERP***: *percent of cell stain pos for ER receptors;* ***ETH***: *ethnicity;* ***FAM***: *family history of cancer;* ***GRA***: *grade of disease;* ***HER***: *HER2 expression;* ***HI1***: *tumor histology;* ***HI2***: *tumor histology subtypes;* ***INL***: *where invasive tumor is located;* ***INV***: *whether tumor is invasive;* ***LYP***: *number of positive lymph nodes;* ***LYR***: *number of lymph nodes removed;* ***LYS***: *patient had any positive lymph nodes;* ***MEN***: *inferred menopausal status;* ***MRI***: *MRIs within 60 days of surgery;* ***NTN***: *number of nearby cancerous lymph nodes;* ***PR***: *progesterone receptor expression;* ***PRP***: *percent of cell stain pos for PR receptors;* ***P53***: *whether P53 is mutated;* ***RAC***: *race;* ***REE***: *removal of an additional margin of tissue;* ***SID***: *side of tumor;* ***SIZ***: *size of tumor in mm;* ***SMO***: *smoking;* ***STA***: *composite of size and # positive nodes;* ***SUR***: *whether residual tumor;* ***TNE***: *triple negative status in terms of patient being ER, PR, and HER2 negative;* ***TTN***: *prime tumor stage in TNM system.*

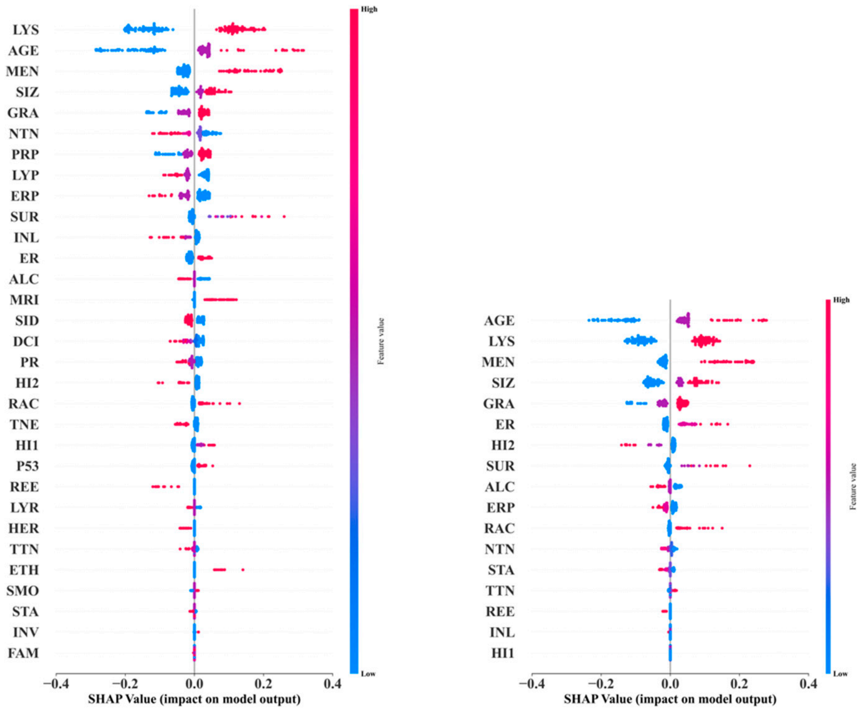

(a) **(b)**

Figure S10. SHAP summary plots for the two best *Logistic Regression* models concerning 15-year BCM (a) LR-15Year; and (b) LR_RF-15Year

AGE*: age at diagnosis of the disease;* ***ALC****: alcohol usage;* ***DCI****: type of ductal carcinoma in situ;* ***ER****: estrogen receptor expression;* ***ERP****: percent of cell stain pos for ER receptors;* ***ETH****: ethnicity;* ***FAM****: family history of cancer;* ***GRA****: grade of disease;* ***HER****: HER2 expression;* ***HI1****: tumor histology****;*** ***HI2****: tumor histology subtypes;* ***INL****: where invasive tumor is located;* ***INV****: whether tumor is invasive;* ***LYP****: number of positive lymph nodes;* ***LYR****: number of lymph nodes removed;* ***LYS****: patient had any positive lymph nodes;* ***MEN****: inferred menopausal status;* ***MRI****: MRIs within 60 days of surgery****;*** ***NTN****: number of nearby cancerous lymph nodes;* ***PR****: progesterone receptor expression;* ***PRP****: percent of cell stain pos for PR receptors;* ***P53****: whether P53 is mutated;* ***RAC****: race;* ***REE****: removal of an additional margin of tissue;* ***SID****: side of tumor;* ***SIZ****: size of tumor in mm;* ***SMO****: smoking;* ***STA****: composite of size and # positive nodes;* ***SUR****: whether residual tumor;* ***TNE****: triple negative status in terms of patient being ER, PR, and HER2 negative;* ***TTN****: prime tumor stage in TNM system.*

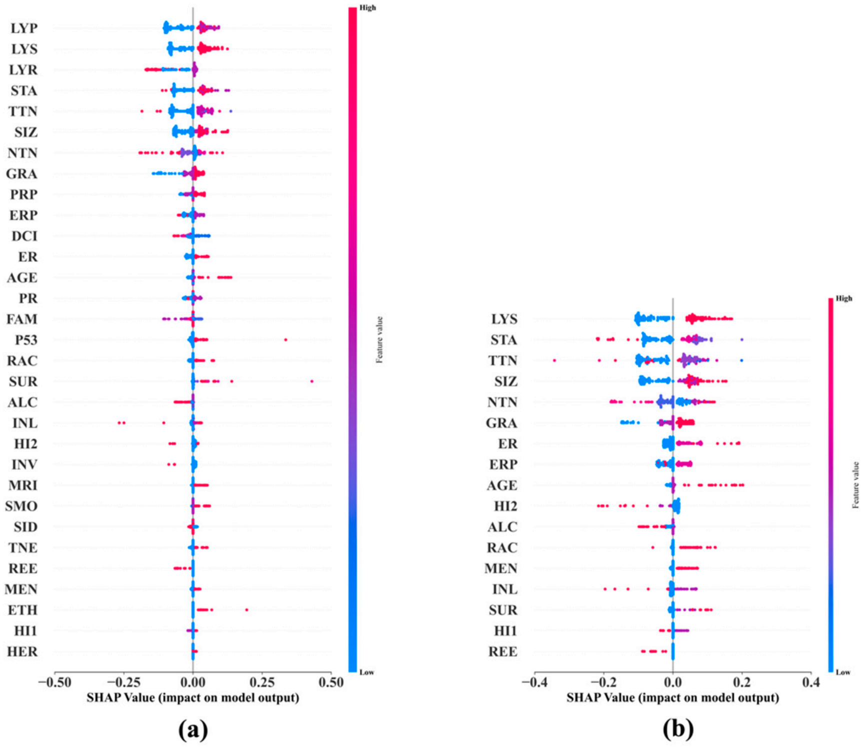

Figure S11. SHAP summary plots for the two best SVC models concerning 15-year datasets
(a) SVC-15Year; and (b) SVC_RF-15Year

AGE: *age at diagnosis of the disease;* ***ALC***: *alcohol usage;* ***DCI***: *type of ductal carcinoma in situ;* ***ER***: *estrogen receptor expression;* ***ERP***: *percent of cell stain pos for ER receptors;* ***ETH***: *ethnicity;* ***FAM***: *family history of cancer;* ***GRA***: *grade of disease;* ***HER***: *HER2 expression;* ***HI1***: *tumor histology;* ***HI2***: *tumor histology subtypes;* ***INL***: *where invasive tumor is located;* ***INV***: *whether tumor is invasive;* ***LYP***: *number of positive lymph nodes;* ***LYR***: *number of lymph nodes removed;* ***LYS***: *patient had any positive lymph nodes;* ***MEN***: *inferred menopausal status;* ***MRI***: *MRIs within 60 days of surgery;* ***NTN***: *number of nearby cancerous lymph nodes;* ***PR***: *progesterone receptor expression;* ***PRP***: *percent of cell stain pos for PR receptors;* ***P53***: *whether P53 is mutated;* ***RAC***: *race;* ***REE***: *removal of an additional margin of tissue;* ***SID***: *side of tumor;* ***SIZ***: *size of tumor in mm;* ***SMO***: *smoking;* ***STA***: *composite of size and # positive nodes;* ***SUR***: *whether residual tumor;* ***TNE***: *triple negative status in terms of patient being ER, PR, and HER2 negative;* ***TTN***: *prime tumor stage in TNM system.*

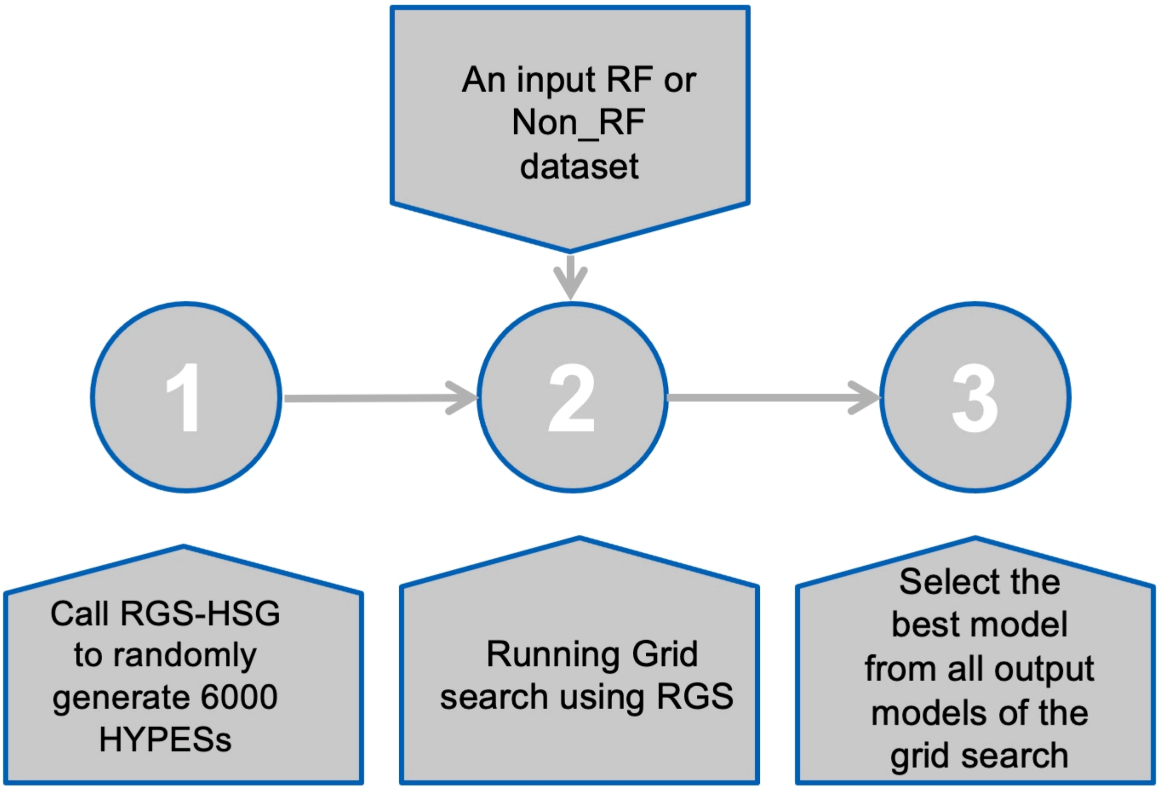

Figure S12. Procedure chart for identifying a best model through grid search.
Note that this procedure will be repeated for each different time horizon of each different ML method.

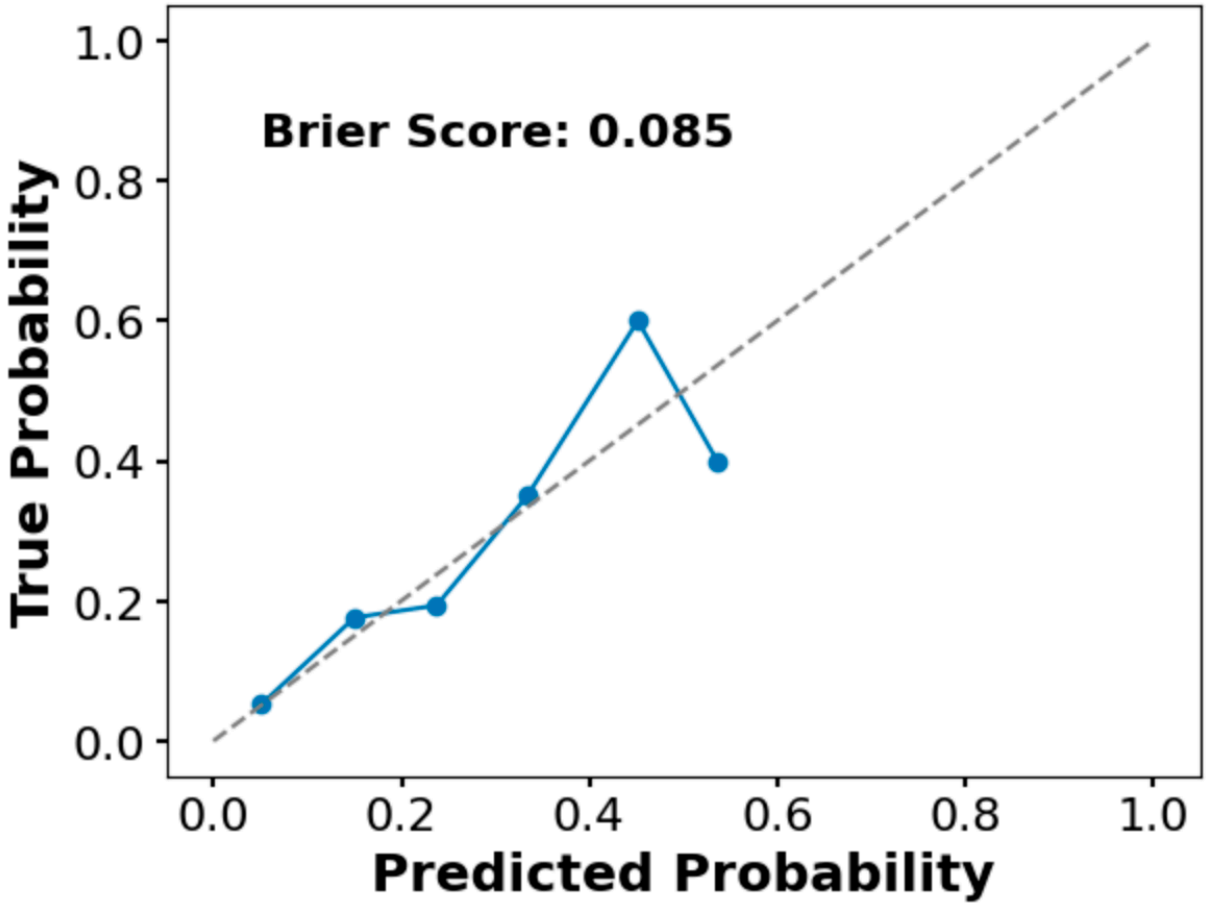

Figure S13. The calibration curve and Brier Score for the best 5_year model (XGB_5Year).

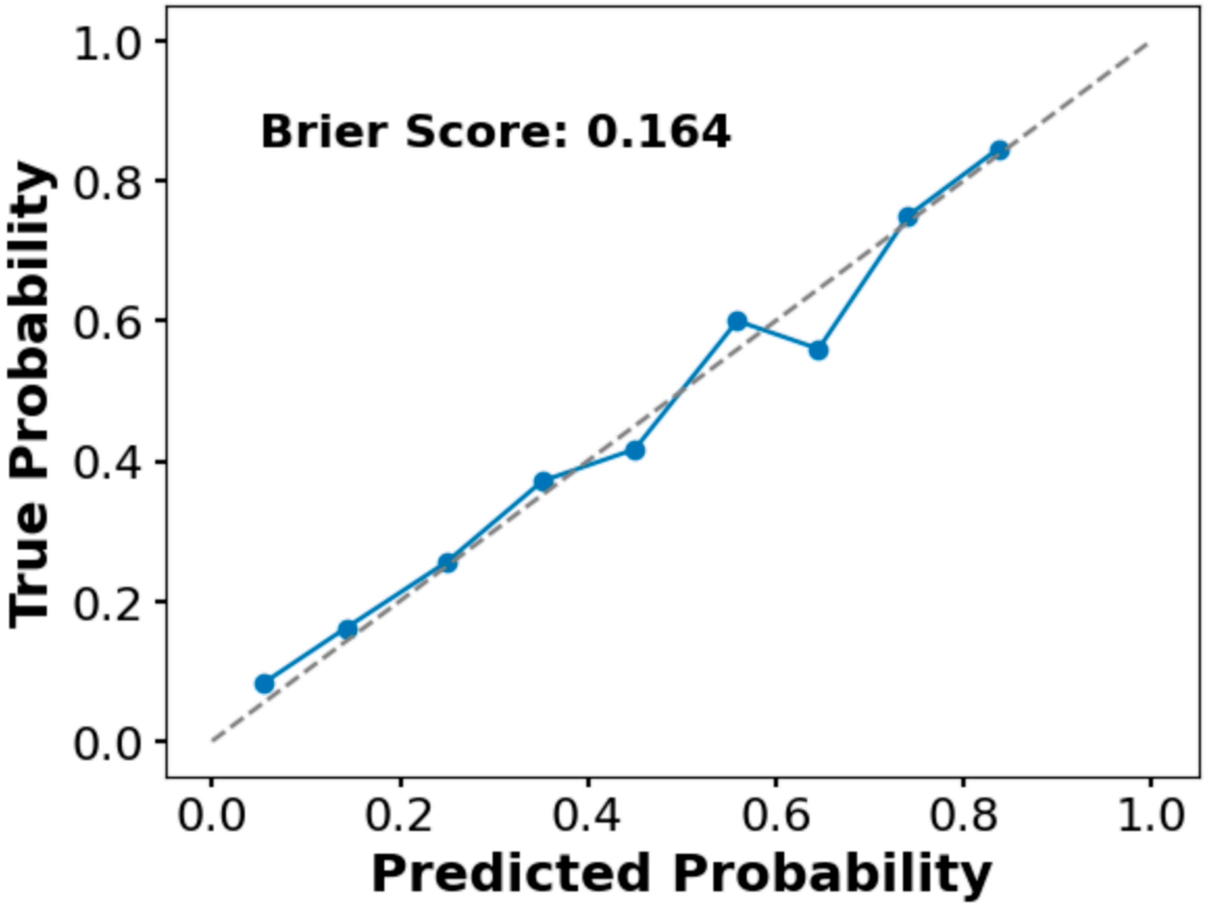

Figure S14. The calibration curve and Brier Score for the best 10_year model (XGB_10Year).

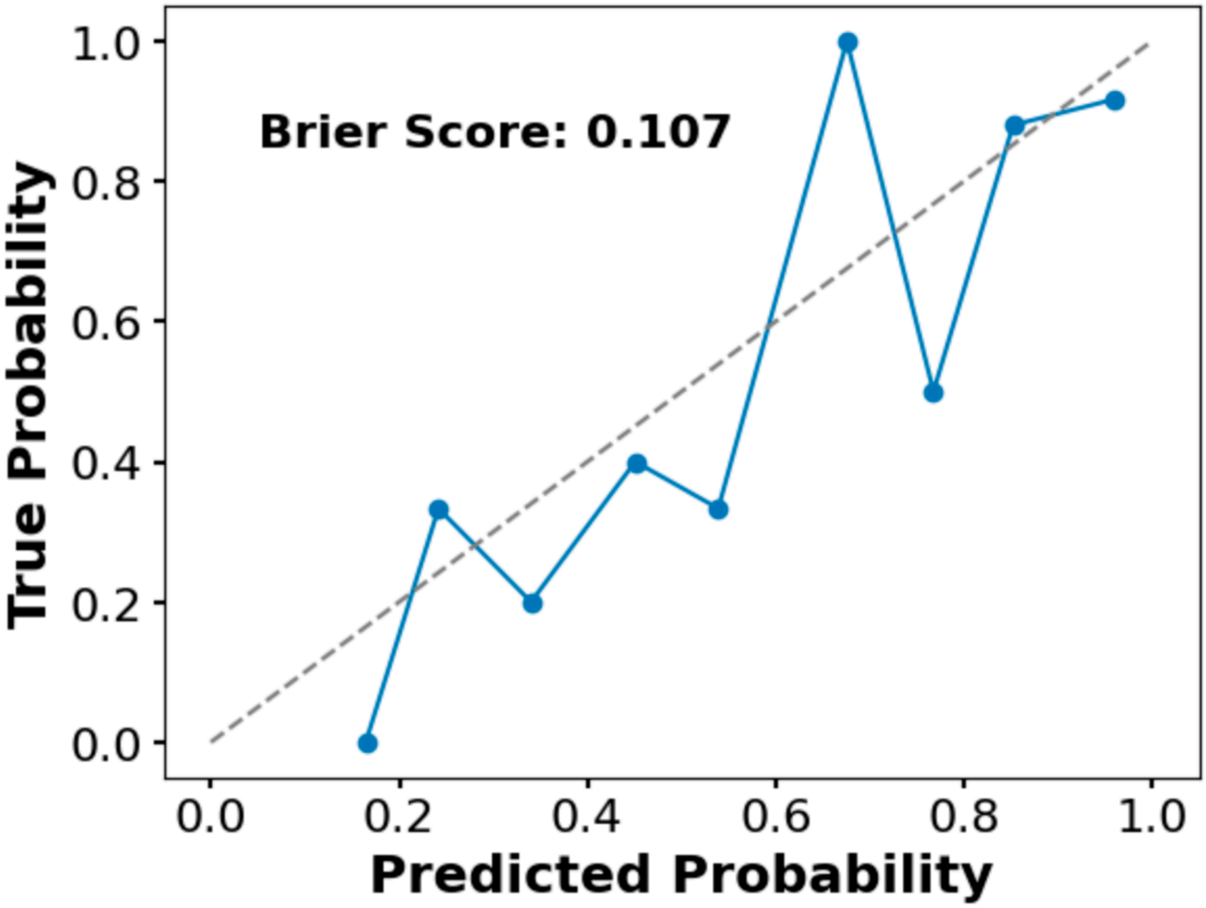

Figure S15. The calibration curve and Brier Score for the best 15_year model (DNM_RF_15Year).
